# Who needs closure? Estimating abundance with a Markovian availability model for geographically open removal sampling

**DOI:** 10.1002/ecy.70289

**Published:** 2026-03-05

**Authors:** Russell W. Perry, Adam C. Pope, A. Noble Hendrix, Joseph E. Kirsch, Bryan G. Matthias, Michael J. Dodrill

**Affiliations:** ^1^ Western Fisheries Research Center, U.S. Geological Survey Cook Washington USA; ^2^ QEDA Consulting, LLC Seattle Washington USA; ^3^ Lodi Fish and Wildlife Office, U.S. Fish and Wildlife Service Lodi California USA; ^4^ Present address: Appalachian Fish and Wildlife Conservation Office, U.S. Fish and Wildlife Service White Sulfur Springs USA

**Keywords:** abundance, availability, density, depletion, geographic closure, N‐mixture, removal, temporary emigration

## Abstract

Removal sampling is an important method for estimating abundance, but nearly all removal models assume closure during sampling. Yet, closure may be difficult to assume, evaluate, or enforce in many settings. To address situations where populations are geographically open between each removal sample, we incorporated a Markovian availability process into an N‐mixture model framework. This model relates local abundance available for sampling to a superpopulation through recruitment of new individuals to the sampling area. To test the model, we (1) conducted parameter identifiability analysis, (2) fit the model to removal data generated from a random walk movement model, and (3) analyzed a case study of empirical removal data. Parameters were increasingly identifiable as capture probability exceeded 0.25 and removal samples increased from 3 to 6. Abundance estimates were unbiased when parameters were identifiable, except for scenarios that simulated a behavioral response to sampling. For our case study, the model estimated negligible recruitment for benthic‐oriented fishes, indicating closure, but we found evidence against closure for juvenile Chinook salmon, a highly mobile species. Our removal model allows researchers to formally test closure assumptions, to estimate the degree of closure, and to estimate abundance without bias when closure is violated.

## INTRODUCTION

Removal models are important tools used to estimate animal abundance and have been applied widely to avian, terrestrial, and aquatic taxa since their first introduction in 1938 (Rodriguez de Rivera & McCrea, 2021). In their simplest form, abundance is estimated by analyzing the geometric decline in successive removals when animals are sampled over multiple occasions at a constant capture probability. Removal models have been extended to accommodate heterogeneity in abundance, to allow for different sampling designs such as continuous‐time sampling, and to account for violations in assumptions such as unequal capture probability among individuals (see review by Rodriguez de Rivera & McCrea, 2021). Closure between sampling occasions is a critical assumption of removal models, as changes in abundance due to births, deaths, immigration, or emigration cause bias in abundance estimates (Fogarty & Fleishman, 2021).

To account for violation of closure, removal models have recently been extended to include open‐population dynamics such as temporary emigration, recruitment, and mortality. The structure of these models depends on whether populations are geographically and demographically open, whether studies estimate abundance at a single sampling site or multiple sites, and whether removal sampling uses a robust design (Pollock, 1982) or allows for an open population between each removal sample (Table 1). Most open‐population removal models employ Pollock's robust design, which combines a closed‐population abundance model for removal samples nested within a primary sampling occasion and an open‐population model for tracking changes in abundance between primary occasions. Only Matechou et al. (2016) allowed for open population dynamics between each removal sample (i.e., a primary sampling occasion consisting of a single removal sample) by using a normal mixture model to account for pulses of recruitment to the local population within the study area.

**TABLE 1:** Characteristics of open‐population removal models.

Publication	Open‐population dynamics	Spatial replication	Sampling design	Model framework
Chandler et al. (2011)	IE: Random availability	Multiple sites	Robust design	N‐mixture model
Zhou et al. (2019)^a^	IE: Markovian availability	Single and multiple sites	Robust design	Hidden Markov Model
This paper	IE: Markovian availability; Assumed stationary local abundance	Multiple sites	Fixed no. primary sampling occasions per site, each with one removal sample	N‐mixture model
Tiberti et al. (2021)	BD: Apparent survival; local recruitment	Single site^b^	Robust design	Population dynamics model
Matechou et al. (2016)	BIDE: Emigration assumed permanent	Single site	Variable no. primary sampling occasions, each with one removal sample^c^	Population dynamics model
Link et al. (2018)	BIDE: Movement among sites; rate of population change	Single study area, Multiple sites^d^	Robust design	Population dynamics model
Udell et al. (2022)	BIDE: Assumed random availability	Single site	Robust design	Population dynamics model

*Note*: “Open‐population dynamics” describes whether the removal model accommodates geographically and/or demographically open populations, the type of open‐population dynamics model used, and notable assumptions or model structure associated with the open‐population dynamics.

Abbreviations: B, birth; D, death; E, emigration; I, immigration.

^a^ Models including survival and models with one removal sample per primary occasions were evaluated, but parameters were found to be non‐identifiable in both cases. Multiple‐site model was generalized to any number of populations but evaluated and applied to a two‐population case.

^b^ Model included multiple sites, but abundance and other model parameters were estimated independently for each site.

^c^ Applications of this model were comprised of consecutive daily removal samples interspersed with variable periods with no sampling ranging from 1 to 12 days over study durations of 2.6–5.2 months.

^d^ Sampling design consisted of removal samples conducted at multiple sites, but this model allowed for movement of individuals among sites and estimated total population abundance over all sites.

The amount of time between primary sampling occasions (which can range from minutes to months) along with the ecology, demography, and behavior of the study subject dictates the structure of open‐population dynamics included in each open removal model. For example, using a robust design for common lizards (*Zootoca vivipara*), Zhou et al. (2019) assumed demographic closure but allowed for temporary emigration between daily primary sampling occasions that each consisted of multiple within‐day removal samples. In contrast, Tiberti et al. (2021) allowed for recruitment and mortality but no immigration or emigration between ice‐free seasons in high‐mountain lakes where closed‐population removal efforts were performed within the ice‐free season to eradicate invasive fishes.

Most open removal models focus on estimating abundance at a single site, but several integrated multiple sites into a single model (Table 1). Link et al. (2018) designed a spatially explicit model that included movement rates among distinct sites to estimate total abundance over the collection of sites comprising the study area. Although their study accommodated multiple sites, interest centered on estimating total abundance over the entire sampling area. Alternatively, Chandler et al. (2011) estimated site‐specific abundance and density by extending closed‐population N‐mixture models to allow for temporary emigration between primary sampling occasions.

Here, we develop an open removal model motivated by sampling geographically open populations at multiple independent sites where closure between successive removal samples cannot be assumed or enforced, ruling out use of a robust design removal model. Further, when removal samples are closely spaced in time, availability of animals for sampling may not be random, but Markovian in nature. That is, an individual within the sampling area on one occasion may be more likely to be available for sampling on the next sampling occasion relative to an individual outside of the sampling area (Ketz et al., 2018). Although several available models tackle at least one of these characteristics (geographically open populations at multiple sites: Chandler et al., 2011; lack of closure between successive removal samples: Matechou et al., 2016; Markovian availability: Zhou et al., 2019), none of the available models include all of these elements.

Therefore, we extend open removal models and show that availability, capture, and abundance parameters are identifiable by incorporating Markovian availability into the hierarchical design of N‐mixture models. N‐mixture models are hierarchical models that use replicated samples (removal sampling, in this case) across space and time to estimate capture probability and abundance (Madsen & Royle, 2023). Variation in abundance among replicates is assumed to arise from a Poisson distribution with an overall mean abundance parameter, and this basic model can be extended to accommodate overdispersion and to include site‐ or time‐specific covariates on abundance. After detailing the model's structure, we test the model by applying it to data generated by the statistical model and to removal data generated using realistic movement of individuals simulated by a two‐dimensional random walk. We then apply the model to benthic fishes and juvenile Chinook salmon (*Oncorhynchus tshawytscha*) collected with beach seines in the San Francisco Bay‐Delta, California, USA.

## METHODS

### Model development

Since our model is an extension of the Chandler et al. (2011) open N‐mixture model, we first briefly describe the N‐mixture sampling protocol, the structure of closed removal models in an N‐mixture framework, and Chandler's extension of closed N‐mixture models to allow for temporary emigration. We then develop the state model for Markovian availability dynamics in the absence of removals. Finally, we detail the observation model that describes the probability of removing individuals, given the Markovian availability state model.

A removal study conducted using an N‐mixture sampling design involves collecting *j* = 1, 2, …, *J* removal samples at *i* = 1, 2, …, *I* sampling units (“sites”), yielding removal counts $y_{i,j}$ (Dorazio et al., 2005). Given closure, removal samples are performed on $N_{i}$, the local abundance at site *i*. The $N_{i}s$ are assumed to follow a Poisson distribution with mean abundance μ, while the removal counts are conditional on $N_{i}$ and follow a multinomial distribution:

(1) $$y_{i,1},y_{i,2},\ldots ,y_{i,J},N_{i}-\sum_{j=1}^{J}y_{i,j}\sim \text{Multinomial}\left(N_{i},\pi_{i,1},\pi_{i,2},\ldots ,\pi_{i,J},\pi_{i,0}\right),$$

where $\pi_{i,j}$ is the multinomial cell probability associated with the probability of first capturing an individual on sample *j*, and $\pi_{i,0}$ is the probability an individual remains uncaptured after *J* removal samples. For a removal‐sampling protocol, the probability of first capturing an individual on sampling occasion *j* is defined as a function of $p_{j}$, the per‐occasion capture probability (dropping the *i* subscript for brevity):

(2) $$\pi_{j}=\frac{\pi_{j-1}}{p_{j-1}}p_{j}\left(1-p_{j-1}\right),j=2,\ldots ,J,$$

and $\pi_{1}=p_{1}$.

This two‐level hierarchical structure forms a multinomial‐Poisson mixture model whose likelihood has a convenient computational form reducing to the product of conditionally independent Poisson distributions (Chandler et al., 2011; Dorazio et al., 2005; Royle, 2004):

(3) $$L\left(\left.\mu ,p\right|y_{i}\right)=\prod_{i}\prod_{j}\text{Poisson}\left(\mu \pi_{i,j}\right).$$

To allow for temporary emigration, Chandler et al. (2011) added a third level to this multinomial N‐mixture model to link a superpopulation of individuals to the local population available for sampling at each site and occasion:

(4) $$\begin{array}{c} M_{i}\sim \text{Poisson}\left(\lambda \right)\\ N_{i,j}\sim \text{Binomial}\left(M_{i},\phi \right), \end{array}$$

where $M_{i}$ is the superpopulation size at site *i* defined as the total number of unique individuals available for sampling, and $N_{i,j}$ is the local abundance available for sampling at site *i* on occasion *j*. The parameter ϕ is the probability that a member of the superpopulation is available for sampling, and λ=μ/ϕ is the mean superpopulation size across sites. In addition to removal sampling, this model can accommodate any multinomial sampling design (e.g., double observer or distance sampling). Chandler et al. (2011) implement this model in a robust design framework because ϕ and p are confounded with only one sample per primary occasion (Table 1). We refer to Chandler's model as the random availability model because $N_{i,j}$ is viewed as a random draw from the superpopulation with availability probability ϕ.

### Markovian availability state model

The random availability model assumes that an individual's availability for sampling on occasion *j* is independent of its availability at occasion *j* − 1, which may be violated if the probability of being available at *j* depends on its prior availability state at *j* − 1 (i.e., available or unavailable). To relax this assumption, we first reframe $M_{i}$ in terms of recruitment of new individuals to the superpopulation and then express ϕ in terms of per‐sample recruitment. Because animals are free to move in and out of the sampling area at site *i* between sampling occasions, let $R_{i,j}$ represent the number of new unique individuals that first enter the sampling area at site *i* on occasion *j*. Thus, $R_{i,j}$ represents recruitment of individuals to the superpopulation at site *i*, which accrues over time at each successive sample occasion:

(5) $$M_{i,j}=\sum_{k=1}^{j}R_{i,k},$$

with $M_{i}=M_{i,J}$ denoting the superpopulation over all *J* samples.

To link $N_{i,j}$ with $M_{i,j}$, we express $N_{i,j}$ as the sum of three components:

(6) $$N_{i,j}=R_{i,j}+M_{i,j-1}^{U\to A}+M_{i,j-1}^{A\to A},$$

where $M_{i,j-1}^{U\to A}$ are the members of the superpopulation that were unavailable (*U*) for sampling at *j* − 1 but re‐enter the sampling area and become available (*A*) by occasion *j*, and $M_{i,j-1}^{A\to A}$ is the number of individuals in the superpopulation that remain available for sampling between samples *j* − 1 and *j*.

We assume that $N_{i,j}$ at site *i* is stationary but fluctuates about a common mean abundance μ over *J* occasions, yielding the following expected values for the components of $N_{i,j}$:

(7) $$\begin{array}{c} E\left[R_{i,j}\right]=\mu \rho_{j}\\ E\left[M_{i,j-1}^{A\to A}\right]=\mu \alpha_{j}\\ E\left[M_{i,j-1}^{U\to A}\right]=\mu \left(1-\alpha_{j}-\rho_{j}\right), \end{array}$$

where $\rho_{j}$ is the probability of a new individual entering the sampling area at site *i* between samples *j* − 1 and *j* measured relative to the mean local abundance, and $\alpha_{j}$ is the proportion of individuals available at *j* − 1 that remain available for sampling on sample *j*. Under the assumption of stationarity, emigrants are balanced by immigrants such that individuals previously in the study area on sampling occasions 1, 2, …, *j* − 2 and unavailable at *j* − 1, re‐enter with probability $1-\alpha_{j}-\rho_{j}$ between *j* − 1 and *j*. This describes a first‐order Markov process, since availability for sampling on occasion *j* depends on the individual's state (available or unavailable) at *j* − 1.

**FIGURE 1:**
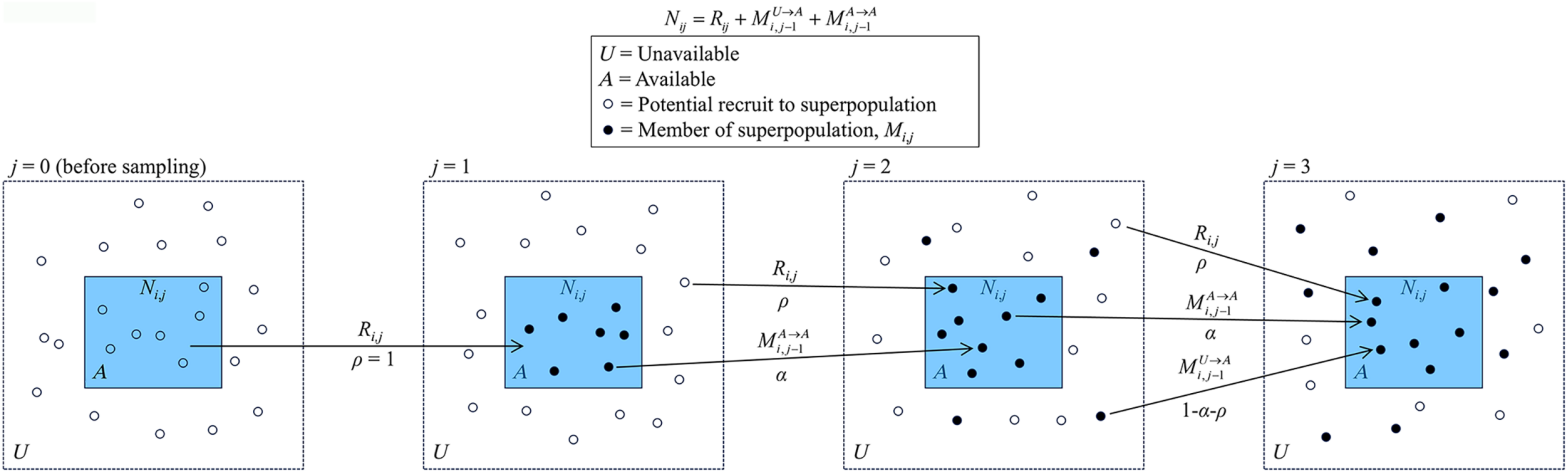
Schematic illustrating state dynamics model with arrows showing state transitions between sampling occasions (*j*) labeled with state variables and transition probabilities. The shaded blue region depicts the sampling area where individuals are available for sampling, with those outside the sampling area being unavailable for sampling. The superpopulation $M_{i,j}$ is defined as the number of unique individuals that were within the sampling area and available for sampling on one or more sampling occasions up to and including sample *j*. Local abundance ($N_{i,j}$) includes individuals that first enter the sampling area on sample *j* and recruit to the superpopulation ($R_{i,j}$), members of the superpopulation that remain available for sampling between *j* − 1 and *j* ($M_{i,j-1}^{A\to A}$), and members of the superpopulation that were unavailable for sampling at *j* − 1, but available at sample *j* ($M_{i,j-1}^{U\to A}$).

This structure provides intuition for how $\rho_{j}$ should vary over *J* occasions. On the first removal sample, all available individuals are new members of the superpopulation, and thus $\rho_{1}=1$ (Figure 1). On the second sample, $M_{i,j-1}^{U\to A}$ = 0 because all individuals entering the sampling area between occasion 1 and 2 are new recruits to the superpopulation. On third and subsequent samples, individuals entering the sampling area will be a mixture of new recruits and members of the superpopulation that had previously occupied the sampling area. Because additions must balance subtractions under the assumption of stationary local abundance, $\rho_{j}$ must decline over each subsequent sampling occasion when $M_{i,j-1}^{U\to A}$ > 0 and individuals are moving in and out of the study area. Otherwise, permanent emigration ($M_{i,j-1}^{U\to A}=0\forall j$) must be balanced by recruitment, which leads to a constant $\rho_{j}$.

Because removal data provide no information about transitions between availability states, let $M_{i,j-1}^{\mathrm{UA}\to A}=M_{i,j-1}^{A\to A}+M_{i,j-1}^{U\to A}$ represent all individuals in the superpopulation at *j* − 1 that are available on sample *j*, irrespective of availability at *j* − 1. The state model now reduces to:

(8) $$N_{i,j}=R_{i,j}+M_{i,j-1}^{\mathrm{UA}\to A}.$$

Because the expected value of $M_{i,j-1}^{\mathrm{UA}\to A}$ is $\mu \left(1-\rho_{j}\right)$, this model structure allows $N_{i,j}$ to be expressed solely in terms of recruitment (ρ) and mean local abundance (μ).

Assuming $R_{i,j}$ is Poisson distributed with mean $\mu \rho_{j}$, then $M_{i}$ is also Poisson distributed:

(9) $$M_{i}\sim \sum_{j=1}^{J}\text{Poisson}\left(\mu \rho_{j}\right)=\text{Poisson}\left(\mu \sum_{j=1}^{J}\rho_{j}\right),$$

where $\rho_{1}=1.$ It is useful here to relate our Markovian availability model to the random availability model. By equating expressions for $E\left[M_{i}\right]$ between the two models (Equation 9 and setting λ=μ/ϕ in Equation 4), we find that the inverse of the availability parameter of the random availability model (ϕ) equates to the sum of the recruitment parameter in our Markovian availability model:

(10) $$\phi^{-1}=\sum_{j=1}^{J}\rho_{j}=E\left(\frac{\lambda }{\mu }\right).$$

This summary statistic represents the ratio of the superpopulation to the mean local abundance, that is, an abundance inflation factor indicating the number of unique individuals available for capture over all *J* occasions relative to the local abundance available on any given occasion. For example, given $\rho_{1}=1$, $\rho_{2,\ldots ,J}$ = 0.1 and *J* = 6, $\phi^{-1}$ = 1.5 indicating that the number of unique individuals available for capture over the course of sampling is 50% greater than the mean abundance available on any given occasion.

Our model is similar to the Markovian temporary emigration models of Kendall et al. (1997) and the “even‐flow” model described by Zhou et al. (2019). These models also constrain transition probabilities such that emigration is balanced by immigration, leading to an average abundance that remains unchanged through time. Our model differs from Zhou et al. (2019) and Chandler et al. (2011) by introducing the superpopulation recruitment parameter, $\rho_{j}$, and by expressing the superpopulation in terms of the per‐sample recruitment, $R_{i,j}$. This formulation recognizes that the superpopulation itself is a function of the number sample occasions, *J*, since the total number of unique individuals available for sampling depends intrinsically on the number of removal samples (Equation 5).

### Observation model for removal sampling

A removal sample performed on $M_{i}$ yields a vector of counts following a multinomial distribution with the same structure as the closed removal model with the exception that $M_{i}$ replaces $N_{i}$ in Equation (1). To express $\pi_{j}$ as a function of per‐sample capture probability, we first define $\pi_{1}=\phi p_{1}$, where ϕ is defined in terms of $\rho_{j}$ (Equation 10). Here, ϕ scales capture probability relative to the superpopulation such that $\pi_{1}$ is the unconditional probability of first capturing $y_{1}$ out of *M* individuals in the superpopulation given *J* sample occasions. First‐capture probability for the remaining sample occasions can then be defined recursively:

(11) $$\pi_{j}=\phi p_{j}\left[\frac{\pi_{j-1}}{\phi p_{j-1}}\left(1-p_{j-1}\right)\left(1-\rho_{j}\right)+\rho_{j}\right].$$

For example, for $\pi_{2}=\phi \left[\left(1-p_{1}\right)\left(1-\rho_{2}\right)p_{2}+p_{2}\rho_{2}\right]$, the term inside the square brackets represents the probability of first capturing an individual conditional on being available for capture on occasion 2, with $p_{2}\rho_{2}$ representing the probability of capturing new recruits and $\left(1-p_{1}\right)\left(1-\rho_{2}\right)p_{2}$ representing the probability of capturing available members of the superpopulation at *j* = 2 less those that were available but captured during the first sampling occasion. As with $p_{1}$, ϕ scales the probability of first capture on occasion 2 relative to all members of the superpopulation. The Markovian availability model also makes use of the integrated multinomial‐Poisson likelihood of Equation (3), with the mean parameter of the Poisson distribution expressed as $\mu \phi^{-1}\pi_{i,j}$, which is the expected value of the number of animals captured on the *j*th removal sample at the *i*th site. As with all N‐mixture models, model parameters can be expressed as functions of site‐level and occasion‐level covariates, and the model can be extended to account for zero inflation and overdispersion.

### Simulation study

We conducted a simulation study to evaluate model performance under a range of scenarios. First, we fit the model to data generated under the Markovian availability removal model to assess model performance when the data are known to arise from the model's structure. Here, we simulated data for either a constant ρ or when $\rho_{j}$ declined with each successive removal sample (see Appendix S2 for details). Second, to assess the bias induced when violating closure, we fit a closed removal model to data generated under the Markovian availability model. Third, we fit the model to removal data simulated by imposing a removal sampling protocol to movement data generated using a two‐dimensional random walk model (Appendix S2). Here, we evaluated the model fit to data generated under three scenarios: (1) when movement direction was circular uniform, (2) when movement was biased in one direction as might occur with migration or advection, and (3) when individuals increased their movement speed as a behavioral response to sampling. These movement model scenarios allowed us to assess how patterns of removal, local abundance, and superpopulation recruitment arise from process‐based movement dynamics, and whether our statistical model could approximate these patterns sufficiently well to estimate model parameters without bias. An example animation illustrating a removal sampling protocol applied to simulated movement data can be found on the ReadMe page of our code repository (Perry et al., 2025; also see Data Availability Statement).

Given these five scenarios and the true parameter values, we simulated removal samples for 1000 datasets each comprised of *I* = 50 sites and *J* = 6 removal samples at four levels of capture probability (p = 0.10, 0.25, 0.5, and 0.75). For each dataset, we then estimated either two parameters (closed removal model), three parameters (constant ρ scenario), or four parameters (all other scenarios): p, μ scaled to sampling area to estimate log‐density ($\theta_{0}$, per square meter), and a linear model for $\text{logit}\left(\rho \right)$ with either an intercept ($\beta_{0}$) for the constant ρ scenario or both an intercept and slope ($\beta_{1}$) for all other models (Appendix S2). Parameters were estimated via maximum likelihood by minimizing the negative log‐likelihood function using the optim routine in R. For all simulations, we examined patterns in removal data and assessed the distribution of parameter estimates relative to true values. For data generated under the Markovian availability model, we evaluated parameter identifiability using the definition of “practical identifiability” outlined by Cole (2020) for each dataset and when subsetting the data to *J* = 3 (Appendix S3).

### Case study

We applied the Markovian availability model to a study of near‐shore fishes collected by beach seine in the Sacramento‐San Joaquin River Delta, California, USA (United States Fish and Wildlife Service et al., 2025). The Delta Juvenile Fish Monitoring Program, a multi‐agency monitoring program, has used single‐pass beach seine sampling for over 30 years to provide an index of juvenile fish abundance. To update their sampling protocol to account for imperfect capture, a pilot study investigated the use of removal sampling with and without enclosures (i.e., geographically closed and open, respectively; see Perry et al., 2016 for details). Here, we focus only on the geographically open samples without enclosures. Briefly, the sampling protocol was as follows: Six replicate beach seine samples were conducted consecutively at each site during every sampling occasion using a 5.2 × 1.3‐m beach seine with 3‐mm square mesh, a continuous lead line, foam floats along the top line, and a centered 1.2‐m^3^ bag. Seines were deployed from shore, the start time of each removal sample was recorded, and the sampling area was calculated as the product of the width of the deployed seine and distance from the shore. After each removal sample, individuals were identified to species and enumerated.

We applied the model to two groups: juvenile Chinook salmon and benthic fishes (Lamprey, *Lampetra* spp.; suckers, Catostomidae; gobies, Gobiidae; sculpins, Cottidae; and logperch, *Percina* spp.). We chose these groups of fish because their contrasting swimming modes (free swimming vs. bottom dwelling) may influence recruitment dynamics (i.e., their propensity to move in or out of the sampling area between samples). Of the 48 open removal samples collected between 2013 and 2015, juvenile Chinook salmon were captured in 17 samples and benthic fishes were collected in 24 samples, with total catches ranging from 2 to 589 fish (median = 80 fish) and 1 to 50 fish (median = 2.5 fish), respectively. Although one would typically include observations with zero total catch, we exclude samples with zero catch from our analysis because our goal is to understand patterns of removal in geographically open samples when the species of interest are known to be present. However, we note that our modeling framework could be extended to accommodate data‐generating processes giving rise to zero catch (e.g., zero inflated or hurdle models).

Given the small sample sizes, we first inspected likelihood profiles to verify that parameters were identifiable for each dataset (Appendix S3: Figure S6) and then fit the simplest possible model structure that adequately explained the data. The simplest model estimated a constant ρ, a constant p, and a constant μ that was scaled to sample area to estimate density (per square meter). To allow for extra variability in abundance and detection, we fit Bayesian hierarchical forms of the model that included site‐level random effects on abundance and detection (where “site” refers to *i* = 1, …, *I* sample units) and pass‐level random effects on capture probability (where “pass” refers to the *j*th removal sample at site *i*). We fit six different models that included no random effects (Model 1), site‐level random effects on either abundance or capture probability (Models 2 and 3) or on both terms (Model 4), and both site‐ and pass‐level effects (Model 5).

We also assessed whether $\rho_{j}$ varied with time between removal samples because more individuals can recruit to the sampling area as the time between samples increases. Thus, Model 6 included all three random effects terms in Model 5 but expressed ρ as a function of time between samples, which ranged from 2 to 13 min:

(12) $$\rho_{j}=1-e^{-r\left(t_{j}-t_{j-1}\right)},$$

where *r* is the instantaneous rate of recruitment to the superpopulation, and $t_{j}-t_{j-1}$ is the time between samples *j* − 1 and *j*. This form reveals that as the time between samples tends toward zero, $\rho_{j}$ approaches zero and the model collapses to the standard N‐mixture model for a closed population with a removal sampling protocol.

The full random‐effects model for p was structured using a logit‐normal mixture:

(13) $$\text{logit}\left(p_{i,j}\right)=\gamma_{0}+\varepsilon_{i}+\eta_{i,j},$$

where $\text{logi}t^{-1}\left(\gamma_{0}\right)$ is the mean capture probability, $\text{logi}t^{-1}\left(\gamma_{0}+\varepsilon_{i}\right)$ is the mean capture probability for site *i*, $\varepsilon_{i}$ is the normally distributed deviation of site *i* from the mean capture probability (on the logit scale) with SD $\sigma_{p,\text{site}}$, and $\eta_{i,j}$ is the deviation of seine pass *j* at site *i* from the mean capture probability of site *i* with SD $\sigma_{p,\text{pass}}$. Similarly, random effects for abundance were modeled using a Poisson normal mixture:

(14) $$\log\left(\mu_{i}\right)=\log\left(A_{i}\right)+\theta_{0}+\omega_{i},$$

where $A_{i}$ is the area sampled (in square meters), $\exp\left(\theta_{0}\right)$ is fish density (per square meter), and $\omega_{i}$ is the normally distributed deviation of site *i* from the log‐mean density with mean zero and SD $\sigma_{\mu ,\text{site}}$. Uninformative or weakly informative priors were used for all parameters (Appendix S1: Table S1).

Models were fitted using JAGS (Just Another Gibbs Sampler; http://mcmc-jags.sourceforge.net/) as implemented by the runjags package in R (Denwood, 2016). All models were run with 3 chains for 250,000 iterations each, a burn‐in of 50,000 iterations, and thinning to every 200th iteration for a final posterior distribution of 3000 iterations. Parameters were checked for convergence using the R‐hat statistic. We compared models using the expected log‐pointwise predictive density (ELPPD) from the LOO package (Vehtari et al., 2024) and assessed goodness of fit using an omnibus test that calculated a Bayesian *p* value based on the Tukey–Freeman statistic. Inference was based on models with the lowest ELPPD and *p* values >0.05, indicating adequate fit of the model to the data.

## RESULTS

### Model behavior and parameter identifiability

**FIGURE 2:**
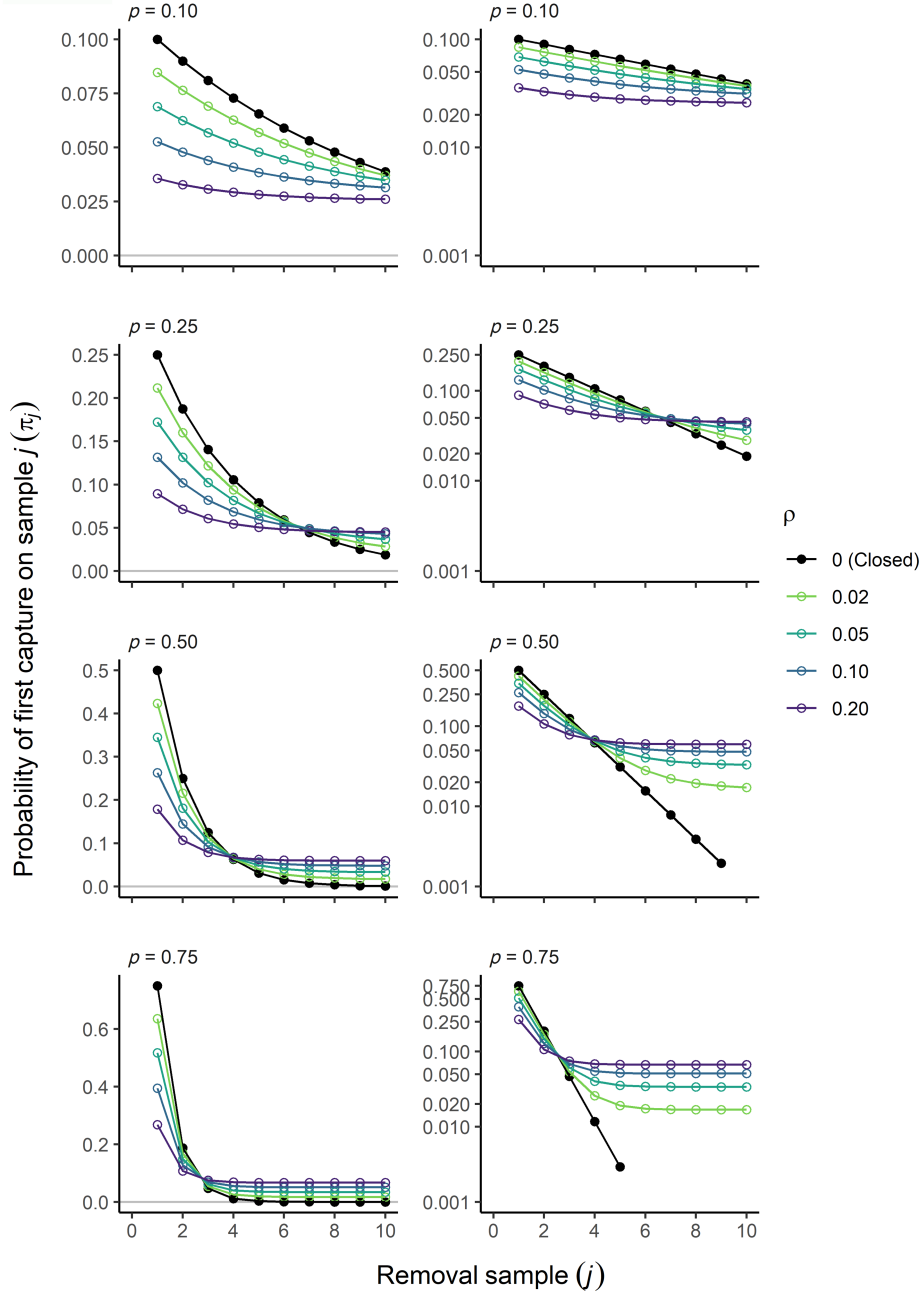
Probability of first capture as a function of the *j*th removal sample, with each panel showing a different capture probability and lines showing different values of ρ. The left column of panels shows the *y*‐axis on the natural scale and the right column displays a logarithmic scale.

Expected patterns in removal data generated by our Markovian model differ markedly from a closed population, with two prominent features emerging (Figure 2). First, $\pi_{j}$ declines more slowly over *j* than a closed population, with the rate of decline decreasing as ρ increases. Second, rather than geometrically approaching zero as *j* approaches infinity, as occurs with the standard closed‐population model, first‐capture probabilities in the Markovian removal model approach a non‐zero asymptote that increases with ρ. These characteristics arise from the interplay of capture probability and recruitment (Equation 8). In the first several samples, $\pi_{j}$ declines more slowly under an open population because as ρ increases, fewer members of the superpopulation are available for sampling on any given occasion relative to a closed population, which reduces the probability of removing an individual from the superpopulation. As animals are depleted and catches decline with subsequent samples, removals are eventually balanced by recruitment, leading to a constant first‐capture probability.

These two features—the initial decline and subsequent leveling off to a non‐zero asymptote—are what allows the additional parameter ρ to be estimated from the removal data without the need for a robust study design, which requires closure between secondary removal samples. Parameter identifiability can be understood graphically by plotting $\pi_{j}$ on a log scale, which highlights the log‐linearity of the closed removal samples versus the initial decline and leveling off in the Markovian availability model. For the closed model, two parameters can be estimated from the straight line, with the slope informing capture probability and the intercept informing abundance (Leslie & Davis, 1939). In contrast, the non‐linearity in log‐space displayed by the Markovian model suggests that three parameters should be estimable, with ρ being the additional parameter related to the asymptote.

Although all parameters should be theoretically estimable, scenarios in which $\pi_{j}$ is approximately log‐linear over the range of *J* hint at the potential for confounding between p and ρ. At low capture probabilities (e.g., p = 0.1), there is no clear transition from an initial depletion signal to a stable removal rate, and the trend is approximately log‐linear over a reasonably large number of removal samples (e.g., *J* = 10). As both p and ρ increase, the asymptote is approached with fewer removal samples, and the distinction between an initial decline and an asymptote becomes more apparent. Yet, even when capture probability is as high as 0.75, an asymptote is not approached until *j* > 3, indicating that more removal samples may be needed to statistically disambiguate p and ρ in a Markovian availability model than would otherwise be required for a closed population removal study.

Formal identifiability analysis applied to simulated datasets supported our insights based on graphical analysis (Appendix S3). Dome‐shaped likelihood profiles with clear maxima for all parameters prove identifiability, whereas profiles that are flat across a wide range of parameter space indicate non‐identifiability of one or more parameters. For the constant‐ρ simulation with *J* = 6, parameters were identifiable for all capture probability scenarios except p = 0.1 (Appendix S3: Figure S2). However, if the simulated data are subset to only three removal samples (*J* = 3), parameters were non‐identifiable for p ≤ 0.25, but otherwise estimable (Appendix S3: Figure S3). For the declining‐ρ scenario, which requires estimation of both a slope and intercept for ρ, parameters were identifiable for *p* ≥ 0.5 with *J* = 6 (Appendix S3: Figure S4), but when *J* = 3, parameters were non‐identifiable for all scenarios, even *p* = 0.75 (Appendix S3: Figure S5).

### Simulation study

**FIGURE 3:**
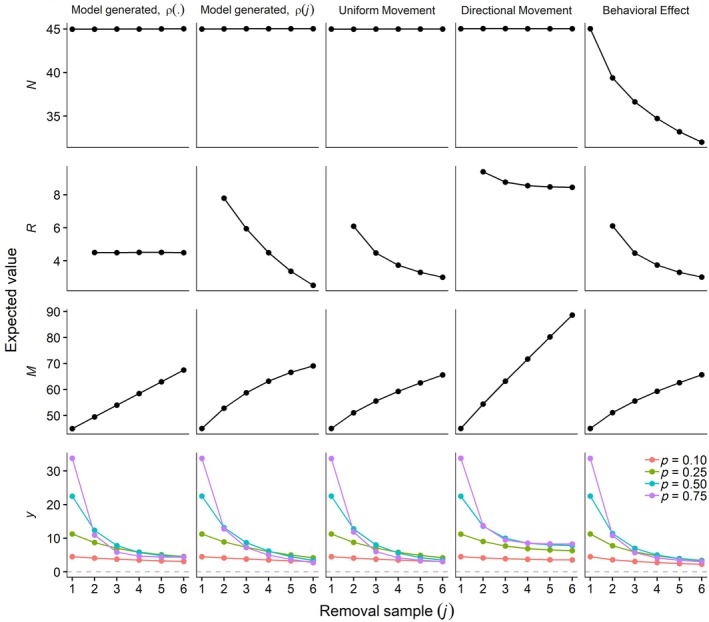
Expected values of local abundance (*N*), recruitment (*R*), superpopulation size (*M*), and removals (*y*) under five different simulation scenarios. To evaluate the assumption of stationary local abundance, *N* shows the average abundance on each sampling occasion in the absence of removals. $R_{1}$ is omitted to focus on the recruitment pattern after the first sample, noting that $R_{1}=N_{1}=M_{1}$. “Model generated” indicates data generated by the Markovian availability removal model with $\rho \left(.\right)$ indicating constant recruitment and $\rho \left(j\right)$ indicating recruitment declining with each removal sample. Expected values were calculated as the mean over all simulations.

The simulations revealed how different movement dynamics gave rise to different patterns in recruitment, local abundance, and removal data (Figure 3). For random uniform movement, recruitment (R) of individuals to the superpopulation declined with each successive removal sample as animals entering the sampling area were increasingly comprised of previously available individuals. In contrast, for directional movement, recruitment was higher and relatively constant owing to a constant influx of new animals and low likelihood of individuals returning to the study area. These differences in movement led to a saturating increase in the superpopulation (M) for random uniform movement but a linear increase for directional movement. Mean local abundance (N) in the absence of removals remained constant except for the behavioral effects scenario. Here, although movement direction was circular uniform, individuals exposed to sampling increased their speed, thereby increasing the rate at which individuals left the study area relative to those that had yet to enter, resulting in a decline in local abundance. Our Markovian availability model was able to generate data that closely matched patterns in removal data generated from realistic movement dynamics. In particular, assuming a declining rate of recruitment (ρ) produced data closely matching that arising from random uniform movement, whereas a constant ρ approximated directional movement. Furthermore, the patterns in removals for different levels of capture probability (Figure 3) closely follow the pattern of first‐capture probabilities under the Markovian removal model (Figure 2), illustrating how removals initially decline before leveling off to a relatively constant number removed.

**FIGURE 4:**
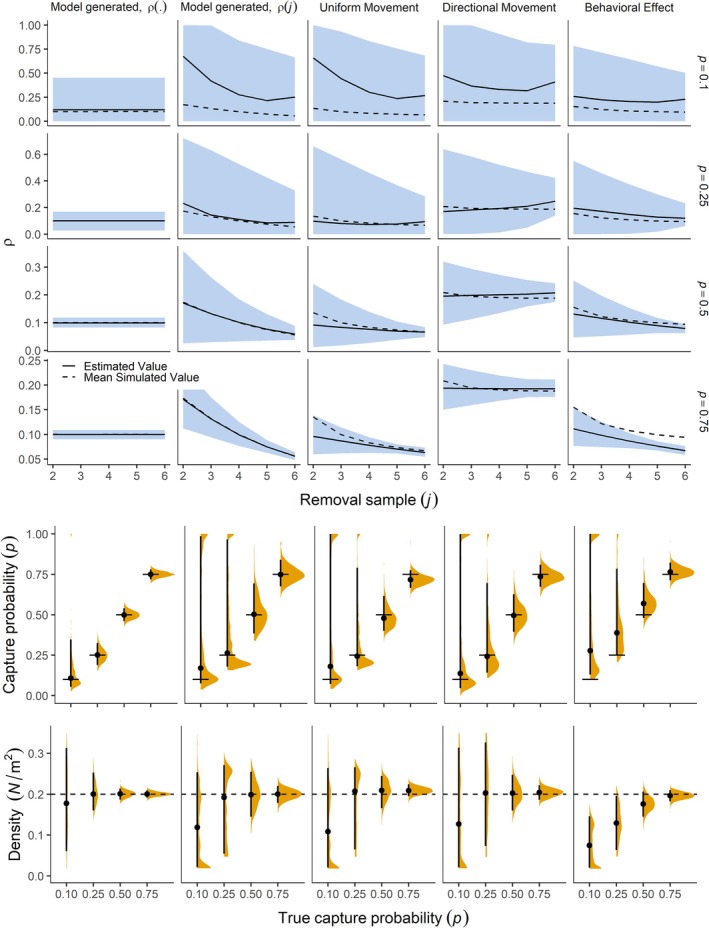
Distribution of maximum likelihood estimates from fitting the Markovian availability removal model to 1000 simulated datasets for each scenario. In the top set of panels, median maximum likelihood estimates of ρ are shown with error bands formed from the 5th and 95th percentile. In the bottom set of panels, the dashed line shows the true value of animal density and horizontal solid lines show the true value of capture probability. Density represents mean local abundance (μ) divided by sampling area (45 m^2^). “Model generated” indicates data generated by the Markovian availability removal model with $\rho \left(.\right)$ indicating constant recruitment and $\rho \left(j\right)$ indicating recruitment declining with each removal sample.

The Markovian availability model was able to accurately estimate abundance and other parameters for most high capture probability scenarios but not when capture probabilities were low (Figure 4). These findings align with our identifiability analysis, generally yielding low bias when parameters were identifiable. The model was unable to estimate any parameter with accuracy for all scenarios with p = 0.1. For p = 0.25, parameter estimates were unbiased for the constant‐ρ scenario, but biased in the declining‐ρ and movement model scenarios (Figure 4). Although the capture probability of 0.25 produced median parameter values close to true or simulated values in all but the behavioral‐effect scenario, the distribution of density estimates from the 1000 simulated datasets was bimodal, with modes on opposite sides of the median, which provided further evidence of non‐identifiability.

In contrast, parameters exhibited little bias for most scenarios when capture probability was greater than 0.5 (Figure 4), except when a closed removal model was fit to data generated by the Markovian availability model (Appendix S2: Figure S2). Here, local abundance was biased high and estimated to be just slightly lower than the true superpopulation size. Median estimated abundance under the closed removal model was inflated by 1.43–1.50 times the true local abundance, whereas the true abundance inflation factor was either 1.5 or 1.54, depending on scenario (Appendix S2: Figure S2). All parameters were estimated accurately when removal data were generated from the Markovian availability model and the directional movement model. For random uniform movement, the general form of the logit link function provided an imperfect fit to the pattern in ρ simulated by the movement model, underestimating ρ in the first few removal samples. However, animal density was estimated accurately, with a relative bias of <5%. For the behavioral effect model, capture probability and density were biased for all but the p = 0.75 scenario. For this scenario, such a high proportion of individuals were removed on each sample that few uncaptured individuals likely remained to influence parameter estimates. For the lower capture probability scenarios, these findings provide insight about the effect of violating the key assumption that emigration from the sampling area is balanced by immigration.

### Case study

Patterns in the removal data, particularly for Chinook salmon, display a steep initial depletion signal followed by a stable, low level of catch thereafter (Appendix S1: Figure S1). This pattern was similar to simulated open removal data (Figure 3) and consistent with the pattern of first‐capture probabilities of the Markovian availability model (Figure 2), providing evidence of recruitment of new individuals to the sampling area.

The removal data also indicate the presence of both site‐ and pass‐level variation in catch (Appendix S1: Figure S1). Model selection and goodness‐of‐fit tests support these observations. For both datasets, only the two most complex models with all random‐effects terms (Models 5 and 6) provided adequate fit to the data, and these models had the lowest ELPPD values (Appendix S1: Table S2). The simplest model with no random effects (Model 1) was ranked lowest—adding either site‐ or pass‐level random effects to this model (Models 2–4) substantially lowered ELPPD. The top two models included all random effects and either constant or time‐dependent recruitment. Although time‐dependent recruitment (Model 6) was the lowest‐ELPPD model for both Chinook salmon and benthic fishes, the difference in ELPPD between the top two models was small and bracketed by the estimated uncertainty.

Both mean capture probability and mean density were lower for benthic fishes relative to Chinook salmon. The posterior median of mean capture probability for benthic fishes was 0.198 compared to 0.368 for Chinook salmon (based on Model 6; Table 2). The posterior median density was 0.131 m^−2^ for benthic fishes and 0.682 m^−2^ for Chinook salmon (based on Model 6; Table 2). Site‐level mean densities varied over 2 and 3 orders of magnitude, respectively, for benthic fishes and Chinook salmon (Appendix S1: Figure S2). Pass‐level estimates of capture probability revealed a pattern where most sites displayed a decline in capture probability of Chinook salmon between the first and subsequent passes (Appendix S1: Figure S3). This pattern was less pronounced for benthic fishes.

**TABLE 2:** Summaries of posterior distributions for parameters of the top two competing models.

Group	Parameter	Model 5		Model 6	
		Median (95% HPDI)	Mean (SD)	Median (95% HPDI)	Mean (SD)
Chinook salmon	$\gamma_{0}$	−0.391 (−1.662 to 0.850)	−0.384 (0.621)	−0.540 (−1.654 to 0.418)	−0.552 (0.531)
	$\text{logi}t^{-1}\left(\gamma_{0}\right)$	0.403 (0.151–0.689)	0.412 (0.136)	0.368 (0.161–0.603)	0.373 (0.116)
	$\sigma_{p,\text{site}}$	1.424 (0.393–2.564)	1.462 (0.554)	1.298 (0.374–2.485)	1.330 (0.523)
	$\sigma_{p,\text{pass}}$	0.917 (0.641–1.480)	0.973 (0.238)	0.919 (0.650–1.317)	0.953 (0.188)
	$\theta_{0}$	−0.355 (−1.221 to 0.673)	−0.327 (0.483)	−0.383 (−1.290 to 0.545)	−0.378 (0.467)
	$\exp\left(\theta_{0}\right)$	0.701 (0.235–1.714)	0.815 (0.451)	0.682 (0.182–1.488)	0.764 (0.375)
	$\sigma_{\mu ,\text{site}}$	2.057 (1.425–2.876)	2.105 (0.376)	2.063 (1.407–2.810)	2.104 (0.367)
	ρ	0.021 (0.005–0.094)	0.029 (0.024)	NA	NA
	*r*	NA	NA	0.205 (0.012–0.710)	0.254 (0.201)
Benthic fishes	$\gamma_{0}$	−1.372 (−3.266 to 0.128)	−1.433 (0.870)	−1.401 (−3.189 to 0.125)	−1.453 (0.849)
	$\text{logi}t^{-1}\left(\gamma_{0}\right)$	0.202 (0.017–0.474)	0.223 (0.132)	0.198 (0.021–0.464)	0.219 (0.129)
	$\sigma_{p,\text{site}}$	2.233 (1.229–3.409)	2.275 (0.563)	2.218 (1.185–3.285)	2.246 (0.541)
	$\sigma_{p,\text{pass}}$	0.492 (0.007–1.029)	0.514 (0.313)	0.493 (0.025–1.034)	0.517 (0.289)
	$\theta_{0}$	−2.032 (−2.909 to −1.115)	−2.021 (0.456)	−2.032 (−2.914 to −1.084)	−2.019 (0.469)
	$\exp\left(\theta_{0}\right)$	0.131 (0.045–0.291)	0.147 (0.072)	0.131 (0.040–0.304)	0.149 (0.076)
	$\sigma_{\mu ,\text{site}}$	1.461 (0.841–2.163)	1.483 (0.342)	1.476 (0.902–2.191)	1.498 (0.338)
	ρ	0.004 (0.000–0.018)	0.007 (0.011)		
	r			0.046 (0.000–0.191)	0.067 (0.085)

*Note*: Parameter definitions: $\gamma_{0}$, logit‐scale mean capture probability; $\text{logi}t^{-1}\left(\gamma_{0}\right)$, mean capture probability; $\sigma_{p,\text{site}}$, SD of site‐level random effects on capture probability; $\sigma_{p,\text{pass}}$, SD of pass‐level random effects on capture probability; $\theta_{0}$, logarithm of mean fish density (per square meter); $\exp\left(\theta_{0}\right)$, mean fish density (per square meter); $\sigma_{\mu ,\text{site}}$, site‐level random effects on abundance; ρ, probability of recruiting to the superpopulation between sample *j*‐1 and *j*; *r*, instantaneous rate of recruitment to the superpopulation between sample *j* − 1 and *j*.

Abbreviation: HPDI, highest posterior density interval; NA, not applicable.

**FIGURE 5:**
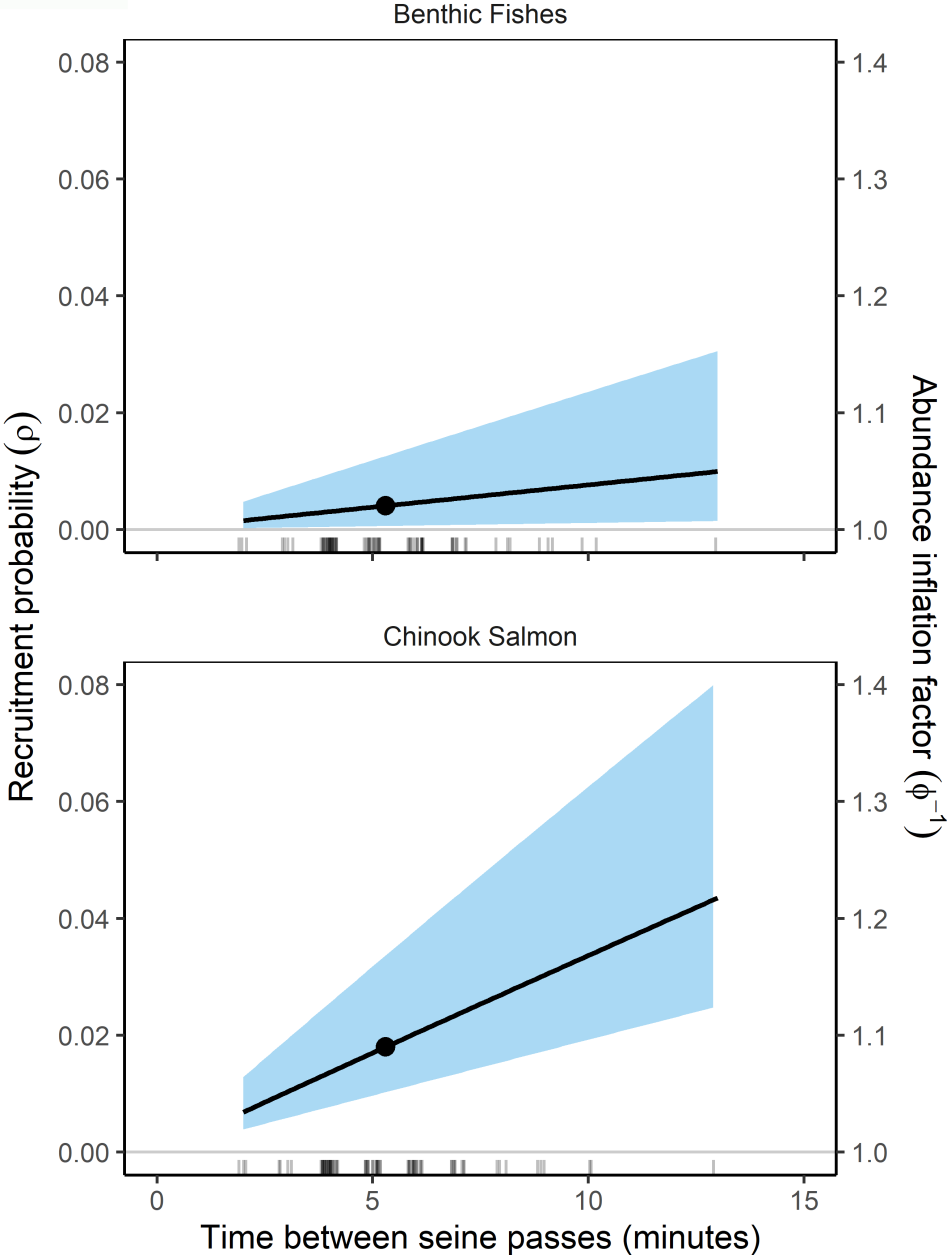
Effect of time between samples on recruitment to the study area (primary *y* axis) and on the abundance inflation factor (secondary *y* axis), expressed as the ratio of the superpopulation abundance to local abundance. The point shows the average time between removal samples, and the rug plot shows the distribution of time between samples (jittered to reduce overlap).

Estimates of recruitment differed between species, providing evidence of closure for benthic fishes but not for Chinook salmon (Table 2). From Model 5, the posterior median of ρ for benthic fishes was 0.004, and the 95% credible interval (CI) contained zero (0.000–0.018). In contrast, for Chinook salmon, the posterior median of ρ was 0.021, and the credible interval excluded zero (95% CI: 0.005–0.094). Similarly, from Model 6, the 95% CI for the instantaneous rate of recruitment (r) bracketed zero for benthic fishes (0.000–0.191) but not for Chinook salmon (0.012–0.710), providing evidence that recruitment of new Chinook salmon to the sampling area increased with time between samples. For example, ρ increased from <0.01 to about 0.04 as the time between samples increased from 2 to 13 min (Figure 5). Across the six removal samples, these findings illustrate how abundance estimates of Chinook salmon could be inflated by between 4% and 20% (Figure 5) by using a removal model that assumed closure. However, realized bias may be slightly lower than indicated by abundance inflation factors since our simulation study identified slightly lower bias for the closed removal model than indicated by the abundance inflation factor.

## DISCUSSION

Most open removal models employ a robust design for estimating detection, availability, and abundance parameters. Without closure between removal samples, parameters are unidentifiable if either availability is assumed random under an N‐mixture study design (Chandler et al., 2011) or if availability is Markovian at a single study site (Zhou et al., 2019). In contrast, when availability is Markovian and cast in the framework of N‐mixture models, we show that recruitment, capture probability, and abundance can be estimated without any closure requirements. In many types of field sampling, geographic closure may be difficult to assume, to measure, or to enforce. Our model offers a potential approach for explicitly accounting for lack of closure, for estimating the degree of closure, and for estimating potential bias associated with applying models that assume closure. On the downside, our Markovian availability removal model requires relatively high capture probabilities and more removal samples than might otherwise be required with a closed population.

Our simulations highlight how animal movement interacts with sampling design to produce different removal patterns that can be estimated with our model. In turn, estimated trends in recruitment provide insights about the nature of movement into and out of the study area. A constant ρ indicates a constant flux of animals moving through the study area with low probability of return visits after departure from the study site, that is, permanent emigration that is balanced by immigration. Such movement dynamics could occur with migration or advection via wind or water currents, such as the juvenile Chinook salmon in our case study which were both migrating seaward and advected by river flows. In contrast, when movement behavior induces temporary emigration, recruitment of new individuals to the sampling area declines with each subsequent sample as local abundance is increasingly comprised of individuals that were previously available for capture. Such patterns may arise with animals moving within their home range, particularly when the sampling area does not fully encompass the home range and sampling frequency allows for movement in and out of the sampling area. Thus, while also accounting for lack of closure, estimated patterns in ρ can provide insights about movement itself.

Considering study design, data collection protocol, and animal movement behavior helps to determine species and settings in which our model is most useful. The spatial extent of the sampling area and time between sampling occasions interact with animal movement to determine availability dynamics. The random availability model may be better suited than our model when animals can fully explore their home range between sampling occasions. In this situation, availability for sampling may be viewed as a random draw from all individuals whose home ranges intersect the sampling area, as Chandler et al. (2011) showed. However, when sampling intervals are of short duration, sampling areas small, and animals mobile, then temporary emigration may not be random but Markovian in nature. Permanent emigration caused by directed movement through the study area is another form of Markovian availability that our model can accommodate.

Removal models are most frequently applied to avian taxa (Rodriguez de Rivera & McCrea, 2021), and our Markovian removal model seems particularly well suited to the subset of avian studies that employ the popular “time‐removal” method (Farnsworth et al., 2002) owing to relatively high detection probabilities (commonly >0.5). The time‐removal method consists of recording first detections of birds within discrete time periods (e.g., *J* = 3) over the course of a minutes‐long point‐count survey (e.g., 10 min). Short time duration and the ability for birds to move in and out of the sampling area between sampling periods could induce Markovian availability dynamics. Farnsworth et al. (2002) discuss how different species may be more or less likely to violate the closure assumption given their territory size and movement dynamics. Our model provides a means to not only formally test this closure assumption but also quantify the degree of closure and avoid potential bias in abundance estimates.

Our analysis showed that more than three removal samples will be needed in most cases; yet, removal studies frequently use only three removal samples. For example, most of the avian studies cited in the review of Rodriguez de Rivera and McCrea (2021) used three removal samples, although several consisted of five or more. Under the Markovian availability model, first‐capture probabilities are approximately log‐linear over the first several removal samples, providing little information to separately estimate both p and ρ (Figure 2). With additional removal samples, expected removals level off or decline more slowly than expected under the geometric decline of a closed removal model, providing the extra information needed to estimate recruitment separately from capture probability. Although parameters were identifiable with *J* = 3 and p ≥ 0.5 under the constant‐ρ simulation scenario, we emphasize that these findings were for “perfect data” that were known to conform with the structure of the Markovian statistical model. In real‐world settings, process variation in abundance, capture, and recruitment argues for more removal samples, not only to ensure parameter identifiability but also to facilitate partitioning of process variation.

Although more sampling effort increases sampling costs, this cost may be low relative to the cost of traveling to and from sampling locations. For example, in our case study, increasing sample effort from three to six removal samples increases sampling time by an average of only about 15 min. Similar costs would be expected for the time‐removal method with birds. In these examples, the trade‐off of increased sampling effort is well worth the value that additional samples provide for separately estimating both p and ρ.

Our case study illustrated the flexibility of the model to accommodate issues that often arise with real data, such as modeling extra variation. For this proof‐of‐concept analysis, we chose to keep the analysis as simple as possible by including only non‐zero catches and modeling extra variation through random effects. A more holistic treatment that fully explores ecological drivers of variation in abundance would expand the model to include not only site‐ and pass‐level covariates but also alternative model structures to accommodate zeros (e.g., zero inflated or hurdle models) and unmodeled variation (e.g., negative‐binomial or beta‐binomial forms). Nonetheless, our case study yielded several insights illustrating the model's utility. First, we found that differences in recruitment aligned with the contrasting swimming modes of the species under study. Sampling could be considered closed for benthic fishes with little potential for bias in abundance with a closed removal model. In contrast, we found strong evidence that the sampling area was geographically open for juvenile Chinook salmon. We also illustrated how recruitment increases with time between samples, with abundance inflation factors of about 9% for the average time between samples or up to 20% for longer times for juvenile Chinook salmon (Figure 5).

Our simulation study found that ignoring closure violations caused considerable positive bias in abundance estimates, as should be expected, but the realized bias was slightly less than indicated by abundance inflation factors. Realized bias was slightly less than expected because the closed removal model fitted a geometric removal process to removal data that did not follow a geometric progression (Figure 2). Nonetheless, the abundance inflation factor provides a useful approximation of the maximum potential bias under a closed removal model and an accurate estimate of the superpopulation size relative to local abundance under the Markovian removal model (Appendix S2: Figure S2). For example, in our case study, the abundance inflation factor for benthic fishes was 1.02 for the average time between removal samples and 1.05 for the maximum time between samples, suggesting little potential for bias by using a simpler closed removal model.

An important assumption of our Markovian state dynamics model is that immigration is balanced by emigration, leading to stationary abundance over time in the absence of removals. This assumption allows $N_{i,j}$ to vary from one occasion to another, but it does not allow for a systematic trend in $N_{i,j}$, either increasing or decreasing. We showed that this assumption is valid both for uniformly distributed animals that moved independently and randomly and for animals that moved with a directional bias (Figure 3). Abundance should also remain stationary for animals moving independently and randomly within home ranges that intersect the sampling area. Assumptions about animal movement dynamics that lead to stationary abundance are embodied by several other abundance estimation methods such as spatial capture–recapture models (Royle et al., 2014) and camera trap models (Amburgey et al., 2021; Gilbert et al., 2021) where animals move through an array of detectors. These assumptions include activity centers that are randomly distributed, animals that move randomly, and animals that move independently of one another. In our model, non‐independent movement among individuals, such as schooling or flocking, could cause abundance to increase or decrease as a group of animals passes through the sampling frame over the course of sampling. However, this process is more likely to induce overdispersion within and among sites rather than systematic bias because the trend in abundance would vary among sites (i.e., sometimes increasing, sometimes decreasing), owing to transient stochastic movement dynamics. In contrast, we believe bias was considerable in our behavioral response simulation because animals exhibited a behavioral avoidance response to sampling at every site, which induced a systematic pattern of declining abundance.

Some types of behavioral responses to sampling are known to cause bias in removal‐based abundance estimates (van Poorten et al., 2017), and our model is no exception. Our movement model illustrated how a behavioral response to sampling manifested as a violation in our model's key assumption that local abundance remains stationary over time with only the composition of individuals changing across samples. In this scenario, local abundance declined during sampling owing to exposed individuals departing the study area more quickly than unexposed individuals entered the study area, resulting in positive bias in capture probability and negative bias in density in all but the p = 0.75 scenario. Model structure can accommodate other types of behavioral responses such as trap shyness or happiness (Otis et al., 1978). For example, by allowing p to vary among removal passes, we identified that juvenile Chinook salmon tended to have higher capture probabilities on the first pass relative to subsequent passes, which could indicate a pattern of trap shyness where individuals exposed to sampling had lower capture probability on subsequent samples.

Recent development of open removal models has been spurred by application to invasive species eradications (Link et al., 2018; Tiberti et al., 2021; Udell et al., 2022) and protected species translocations (Matechou et al., 2016; Zhou et al., 2019). Yet, even most of these open removal studies embody closure assumptions with use of the robust design (Table 1). In many other situations, the closure assumption cannot be assumed or enforced, eliminating the robust design as an option. Nonetheless, researchers often assume closure based on study design characteristics (e.g., samples closely spaced in time) or domain expertise on species of interest (e.g., expected movement dynamics). Our Markovian availability model fills a gap in the spectrum of available open removal models, allowing scientists and managers to formally test closure assumptions, to estimate the degree of closure, and to quantify potential bias associated with assuming closure.

## CONFLICT OF INTEREST STATEMENT

The authors declare no conflicts of interest.

## Supporting information


Appendix S1.

Appendix S2.

Appendix S3.

## Data Availability

Data (United States Fish and Wildlife Service et al., 2025) are available from the Environmental Data Initiative (EDI) Data Portal at https://doi.org/10.6073/pasta/818b8bf1d93b81cc7c5124f9df81ce01. Code (Perry et al., 2025) is available in a U.S. Geological survey software release at https://doi.org/10.5066/P13TM4Y8.
