# Supplementary material for: Who needs closure? Estimating abundance with a Markovian availability model for geographically open removal sampling
Source: Ecology. 2026 Mar 5;107(3):e70289. doi: 10.1002/ecy.70289 (PMC12963955; doi:10.1002/ecy.70289)
Supplement: Supplementary file 1 — Appendix S1. [file ECY-107-e70289-s003.pdf]

Ecology

Who needs closure? Estimating abundance with a Markovian availability model for geographically open removal sampling

Russell W. Perry, Adam C. Pope, A. Noble Hendrix, Joseph E. Kirsch, Bryan G. Matthias, and Michael J. Dodrill

Appendix S1: Case Study Supplemental Figures and Tables

Table S1. Prior distributions used for each Markovian availability removal model.

Model	γ_0	$\sigma_{p,\text{site}}$	$\sigma_{p,\text{pass}}$	θ_0	$\sigma_{\mu,\text{site}}$	ρ	r
1	Beta(1, 1)			N(0, 1)		Beta(1, 1)	
2	Beta(1, 1)			N(0, 1)	Half-N(0, 1.5)	Beta(1, 1)	
3	Cauchy(0, 10)	Half-N(0, 1.5)		N(0, 1)		Beta(1, 1)	
4	Cauchy(0, 10)	Half-N(0, 1.5)		N(0, 1)	Half-N(0, 1.5)	Beta(1, 1)	
5	Cauchy(0, 10)	Half-N(0, 1.5)	Half-N(0, 1.5)	N(0, 1)	Half-N(0, 1.5)	Beta(1, 1)	
6	Cauchy(0, 10)	Half-N(0, 1.5)	Half-N(0, 1.5)	N(0, 1)	Half-N(0, 1.5)		Half-N(0, 1)

Note: Parameter definitions: γ_0, logit-scale mean capture probability; $\sigma_{p,\text{site}}$, standard deviation of site-level random effects on capture probability; $\sigma_{p,\text{pass}}$, standard deviation of pass-level random effects on capture probability; θ_0, logarithm of mean fish density (m^{-2}); $\sigma_{\mu,\text{site}}$, site-level random effects on abundance; ρ, probability of recruiting to the superpopulation between sample *j*-1 and *j*; *r*, instantaneous rate of recruitment to the superpopulation between sample *j*-1 and *j*.

Abbreviations: N, normal distribution; Half-N, half-normal distribution truncated at x ≥ 0.

Table S2. Bayesian model selection and goodness of fit statistics comparing alternative removal model fit to each species.

Species	Model number	ELPPD (SE)	ELPPD Difference (SE)	P-value
Chinook salmon	6	-234.3 (28.1)	0.0	0.50
	5	-235.9 (28.7)	-1.5 (2.3)	0.51
	4	-544.8 (204.3)	-310.5 (187.9)	<0.01
	2	-656.0 (208.8)	-421.6 (189.3)	<0.01
	3	-855.9 (292.9)	-621.6 (275.6)	<0.01
	1	-2,795.9 (726.2)	-2,561.5 (718.8)	<0.01
Benthic fishes	6	-162.5 (19.3)	0.0	0.20
	5	-163.1 (19.7)	-0.6 (2.0)	0.20
	4	-165.7 (20.7)	-3.1 (3.1)	0.05
	3	-184.0 (23.0)	-21.4 (9.3)	<0.01
	2	-201.4 (39.9)	-38.8 (31.5)	<0.01
	1	-488.4 (135.1)	-325.9 (125.9)	<0.01

Note: ELPPD difference is the difference in ELPPD from the lowest-ELPPD model. P-value is the omnibus goodness-of-fit test based on the Tukey-Freeman statistic. Model 1, base model with no random effects; Model 2, site-level random effect on abundance; Model 3, site-level random effect on capture probability; Model 4, site-level random effects on abundance and capture probability; Model 5, site-level random effects on abundance and capture probability and pass-level random effects on capture probability; Model 6, same as Model 5 with ρ_j expressed as a function of time between seine passes.

Abbreviations: ELPPD, expected log pointwise predictive density; SE, standard error.

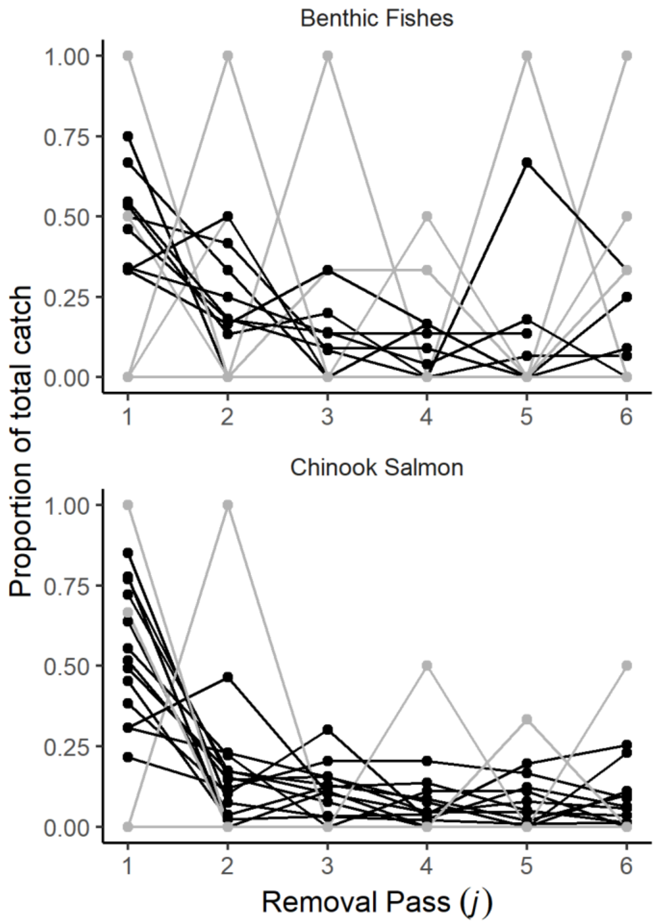

Figure S1. Removal data showing the proportion of the total catch removed on sample *j*. Removal samples with a total catch ≤ 5 are shown as gray lines to minimize the influence of sampling variation on the overall pattern of removals.

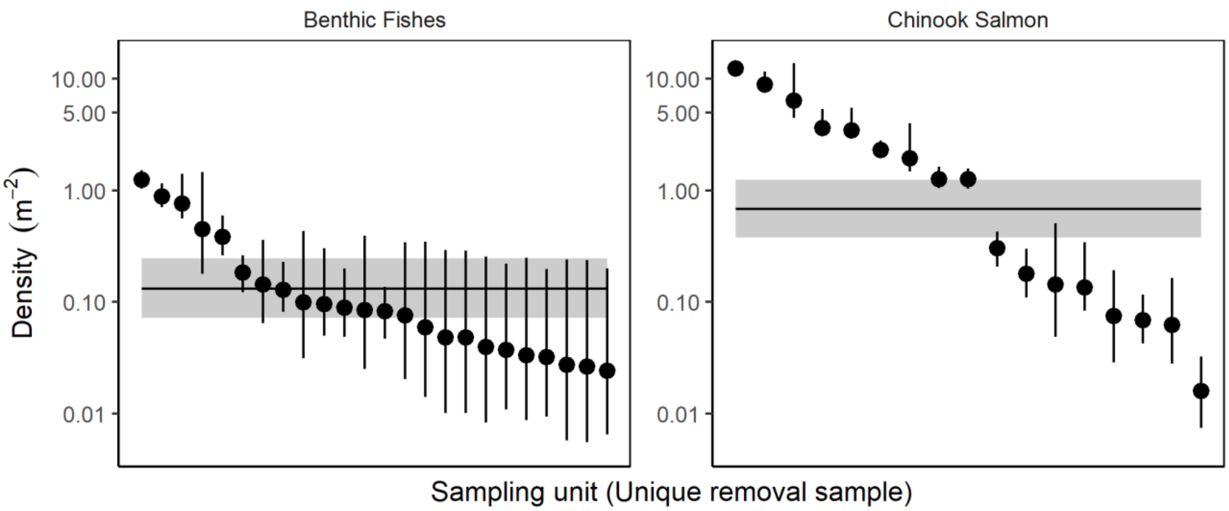

Figure S2. Site-level density estimates for each removal sample with points showing posterior medians and error bars showing 10th and 90th percentiles from Model 6. The horizontal line is the mean density across sites with gray shading showing the 10th and 90th percentiles of the mean density.

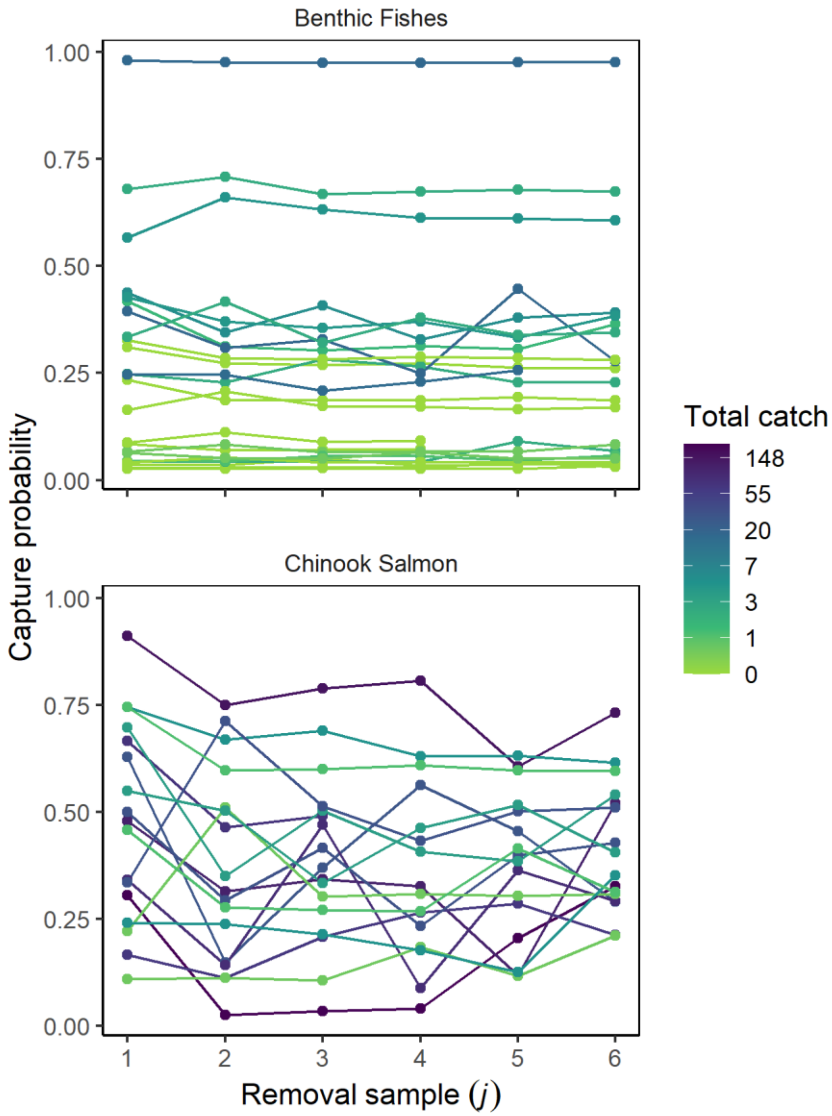

Figure S3. Posterior median detection probability for each site and removal sample.
