# Supplementary material for: Who needs closure? Estimating abundance with a Markovian availability model for geographically open removal sampling
Source: Ecology. 2026 Mar 5;107(3):e70289. doi: 10.1002/ecy.70289 (PMC12963955; doi:10.1002/ecy.70289)
Supplement: Supplementary file 2 — Appendix S2. [file ECY-107-e70289-s001.pdf]

Ecology

Who needs closure? Estimating abundance with a Markovian availability model for geographically open removal sampling

Russell W. Perry, Adam C. Pope, A. Noble Hendrix, Joseph E. Kirsch, Bryan G. Matthias, and Michael J. Dodrill

Appendix S2: Simulation Methods and Supplemental Figures

We designed the simulations to emulate the characteristics of our case study, although our techniques and findings are broadly applicable to a wide range of species, applications, and sampling designs. All simulations used J = 6 removal samples. Simulations expressed abundance as animal density since inference and management often focuses on this metric (Chandler et al. 2011):

$$\log(\mu) = \log(A) + \theta_0 \qquad \text{(S1)}$$

where A is sampling area, set to a 15 x 15-m square in all simulations, and $\exp(\theta_0)$ is animal density which was set to 0.2 individuals / m^2, yielding a mean local abundance of μ = 45 individuals.

For data generated under the Markovian availability removal model, we developed two recruitment scenarios based on recruitment patterns observed in the movement simulations: a constant-ρ scenario were ρ = 0.1, and a scenario where ρ_j declined with each removal sample from $\rho_2 = 0.17$ to $\rho_6 = 0.06$ according to:

$$\text{logit}\left(\rho_j\right) = \beta_0 + \beta_1 j_{\text{std}} \qquad \text{(S2)}$$

where β_1 = -0.5 and j_{std} is the sampling occasion centered on j = 4 and standardized by 1.58 (the standard deviation of j = 2, 3, …, 6) such that β_0 = -2.2 yields an intercept equivalent to the constant-ρ scenario (ρ = 0.1).

For the movement model simulations, we developed a simple two-dimensional random walk model comprised of a Weibull step distribution and a wrapped Cauchy movement direction distribution (McClintock et al., 2012). The simulation area was a 200 x 200-m square, with the sampling frame centered on the origin. Simulations were initialized with a bivariate uniform distribution of spatial locations for each individual, run with a 30-s time step, and removal samples taken at 5-minute intervals. For the base movement model, step lengths at each time step were drawn from a Weibull(1.25, 0.5δ) distribution with δ = 1, and movement direction was drawn from a uniform circular distribution parameterized with a WC(0, 0) distribution (WC = wrapped Cauchy; Figure S1). For the directional movement model, movement direction was drawn from a WC(0, 0.5) distribution (Figure S1). For the behavioral effects model, movement speed doubled (δ = 2) for individuals that were exposed to sampling (Figure S1).

In these movement simulations, the true ρ_j is unknown because it emerges from the underlying movement parameters, the nature of the sampling frame (e.g., size and shape), and the time between removal samples. However, ρ_j can be calculated from the simulations by keeping track of new recruits to the sampling frame ($R_{i,j}$) and dividing by the number of individuals in the sampling frame on each sample ($N_{i,j}$).

References

Chandler, R. B., J. A. Royle, and D. I. King. 2011. "Inference about density and temporary emigration in unmarked populations." *Ecology* 95: 794-794.

McClintock, B. T., R. King, L. Thomas, J. Matthiopoulos, B. J. McConnell, and J. M. Morales. 2012. "A general discrete-time modeling framework for animal movement using multistate random walks." *Ecological Monographs* 82: 335-349.

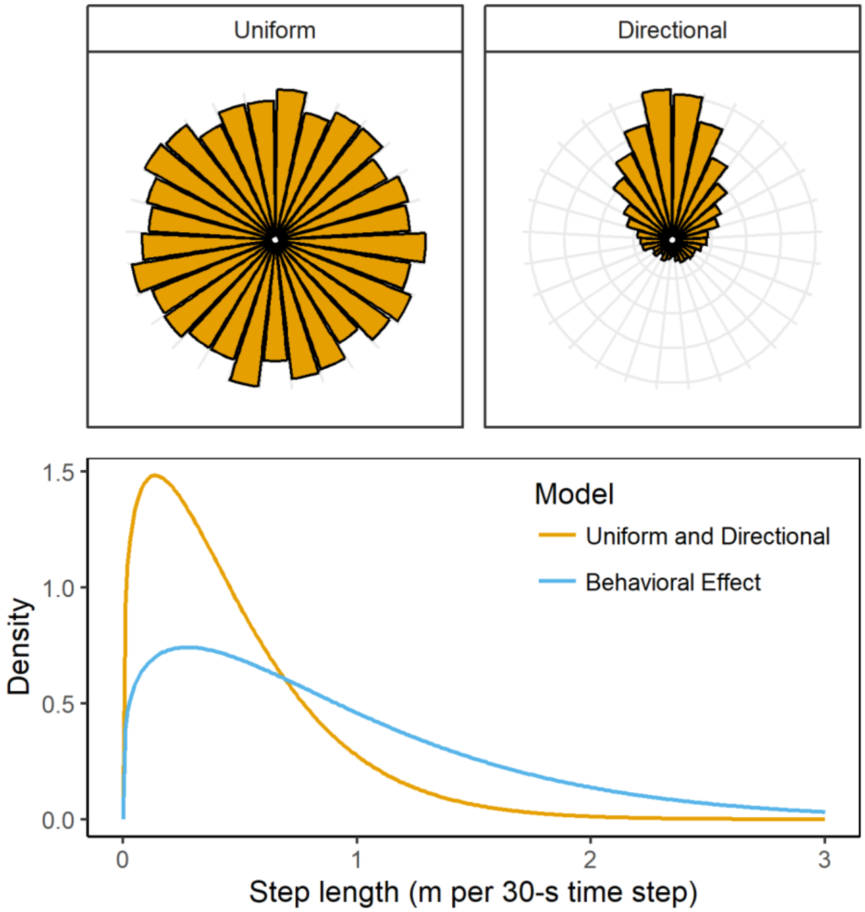

Figure S1. Movement directions (top row) and step-length distributions used to parameterize the three random-walk models: Uniform = uniform circular movement, Directional = movement biased in one direction, and Behavioral Effect = movement speed approximately doubled for individuals exposed to sampling (with uniform movement directions).

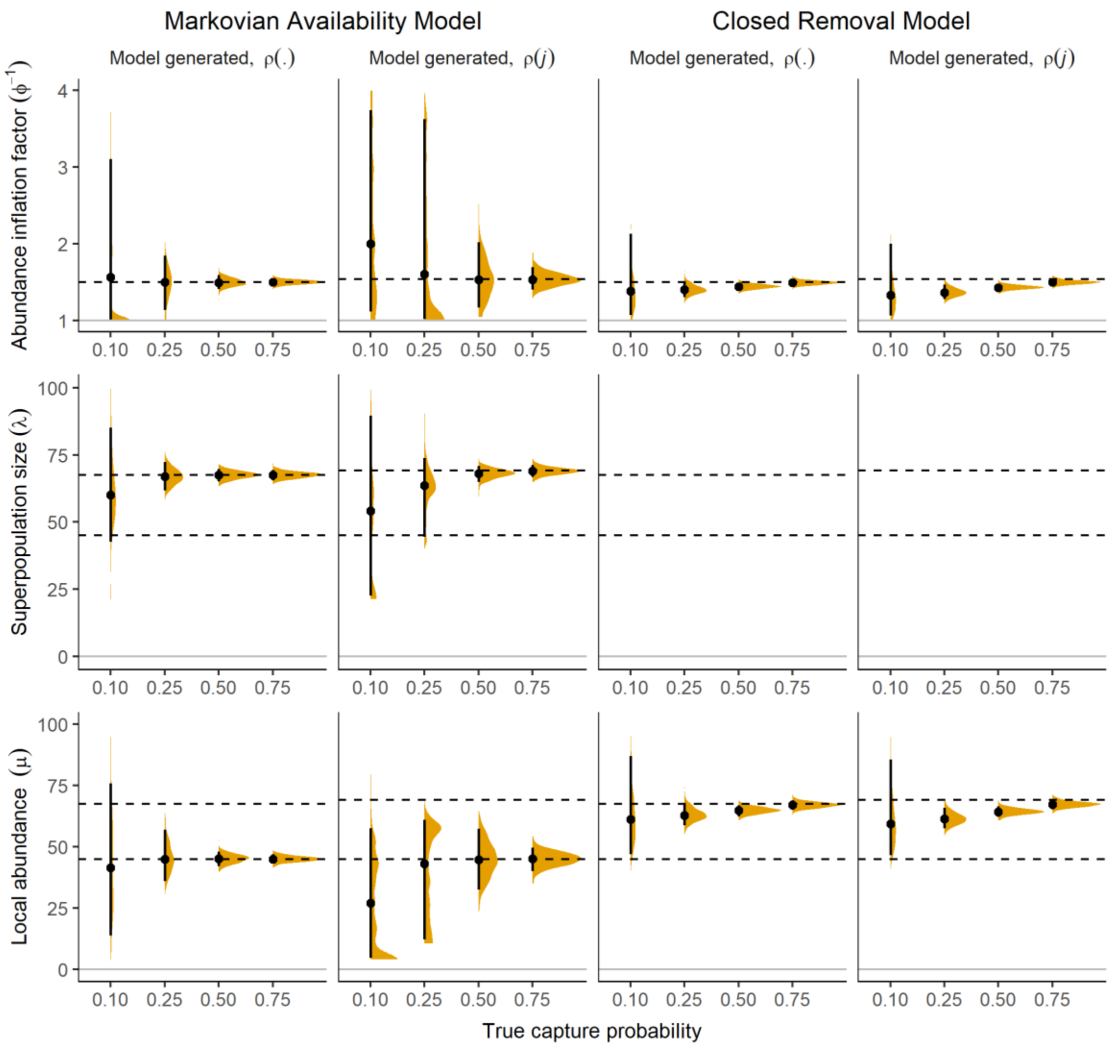

Figure S2. Distribution of maximum likelihood estimates from fitting the Markovian availability removal model and a closed removal model to 1000 simulated data sets for each scenario. In the two bottom rows of panels, the lower dashed line shows the true local abundance and the upper dashed line shows the true superpopulation size. In the top row, the dashed line represents the true abundance inflation factor. For the closed removal model, the abundance inflation factor was calculated by dividing the estimated local abundance by the true local abundance. "Model generated" indicates data generated by the Markovian availability removal model with $\rho(.)$ indicating constant recruitment and $\rho(j)$ indicating recruitment declining with each removal sample.
