# Supplementary material for: Who needs closure? Estimating abundance with a Markovian availability model for geographically open removal sampling
Source: Ecology. 2026 Mar 5;107(3):e70289. doi: 10.1002/ecy.70289 (PMC12963955; doi:10.1002/ecy.70289)
Supplement: Supplementary file 3 — Appendix S3. [file ECY-107-e70289-s002.pdf]

Ecology

Who needs closure? Estimating abundance with a Markovian availability model for geographically open removal sampling

Russell W. Perry, Adam C. Pope, A. Noble Hendrix, Joseph E. Kirsch, Bryan G. Matthias, and Michael J. Dodrill

Appendix S3: Parameter Identifiability

The goal of the parameter identifiability analysis was to determine how characteristics of sampling design interact with true underlying parameter values to affect whether model parameters are estimable. Although all parameters of the Markovian availability removal model are theoretically estimable, aspects of sampling design and underlying true parameter values may give rise to data sets that render model parameters inestimable. This feature is known as extrinsic redundancy, which refers to models in which all parameters are theoretically identifiable but the structure of a particular data set (e.g., missing or sparse data) leads to inability to estimate one or more parameters (Gimenez et al. 2004, Cole 2020).

To determine whether one or more model parameters were inestimable, we used profile likelihood methods and the definition of "practical identifiability" outlined by Cole (2020). A likelihood profile for θ is formed by fixing θ, maximizing the likelihood function with respect to remaining model parameters, repeating over a range of fixed values of θ, and plotting the maximum likelihood value versus θ. A non-identifiable parameter will have a flat likelihood profile whereas a practically unidentifiable parameter will have a maximum but a relatively flat profile (Cole 2022). A parameter is classified as practically unidentifiable if one or both likelihood-based confidence limits do not exist or are infinite over a biologically plausible range of parameter values.

To apply these methods to our simulated data sets, we randomly selected 100 of the 1,000 simulated data sets; constructed likelihood profiles for each parameter, model, and data set; and determined whether both confidence limits existed. A parameter was deemed practically identifiable if its likelihood profile twice crossed the line defined by $l(\hat{\theta}) - 0.5\chi^2_{\alpha,q}$, where $l(\hat{\theta})$ is the log-likelihood evaluated at the maximum likelihood estimate, $\chi^2_{\alpha,q}$ is the quantile function of the chi-squared distribution for a $1-\alpha$ confidence limit with q degrees of freedom, and q is the number of estimated parameters.

We determined practical identifiability for data sets generated by the Markovian availability removal model with a constant ρ and ρ declining over j, with $J = 6$ or the same dataset subset to $J = 3$, and for capture probabilities of 0.10, 0.25, 0.50, and 0.75 (see Appendix S2 for simulation details). We set $\alpha = 0.20$ to determine practical identifiability based on an 80% likelihood-based confidence interval. Profiles were summarized by tabulating the proportion of the data sets where a parameter was classified as identifiable (Figure S1). We also plotted likelihood profiles for each data set, model, and capture probability (Figures S2-S5).

Given the small sample size of our case study data sets ($I = 17$ benthic fishes, $I = 24$ for Chinook salmon) relative to number of samples used for simulation ($I = 50$), we also used likelihood profiles to determine practical identifiability for our case study data sets (Figure S6). We determined practical identifiability for models with either constant ρ or for ρ expressed as a logit-linear function of j. For Chinook salmon, likelihood profiles were dome-shaped, indicating that parameters were estimable for both models. In contrast, for benthic fishes, parameters were estimable for the constant ρ model, but capture probability (p) was classified as unidentifiable for the model that expressed ρ as a function of j. Here, the likelihood profile

was relatively flat for $p > 0.75$ and intersected the upper boundary at $p = 1$. Based on these findings, although the data set for Chinook salmon allowed ρ to be expressed as a function of j, we fit hierarchical Bayesian forms of constant-ρ to both data sets. This allowed us to use a common set of models fitted to both data sets.

References

Cole, Diana. 2020. *Parameter Redundancy and Identifiability*. Boca Raton: CRC Press.

Gimenez, Olivier, Anne Viallefont, Edward A. Catchpole, Remi Choquet, and Byron J.T. Morgan. 2004. "Methods for Investigating Parameter Redundancy." *Animal Biodiversity and Conservation* 27 (1): 561-572.

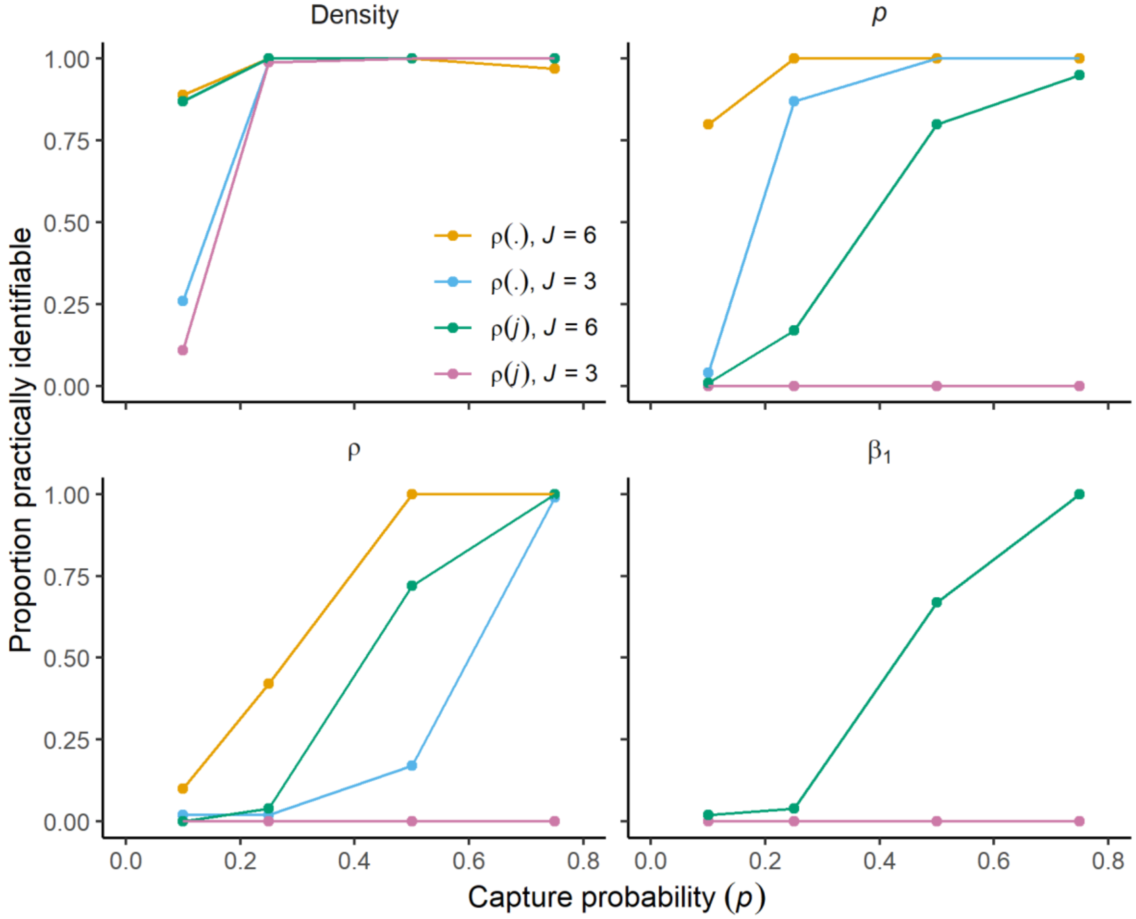

Figure S1. Proportion of simulated data sets in which each parameter is classified as practically identifiable based on an 80% likelihood-based confidence interval. Data sets and models fit to the data include either a constant- ρ model (labeled as $\rho(.)$) or ρ declining with each removal sample (labeled as $\rho(j)$) with J = 6 removal samples or the same simulated data sets subset to J = 3 removal samples.

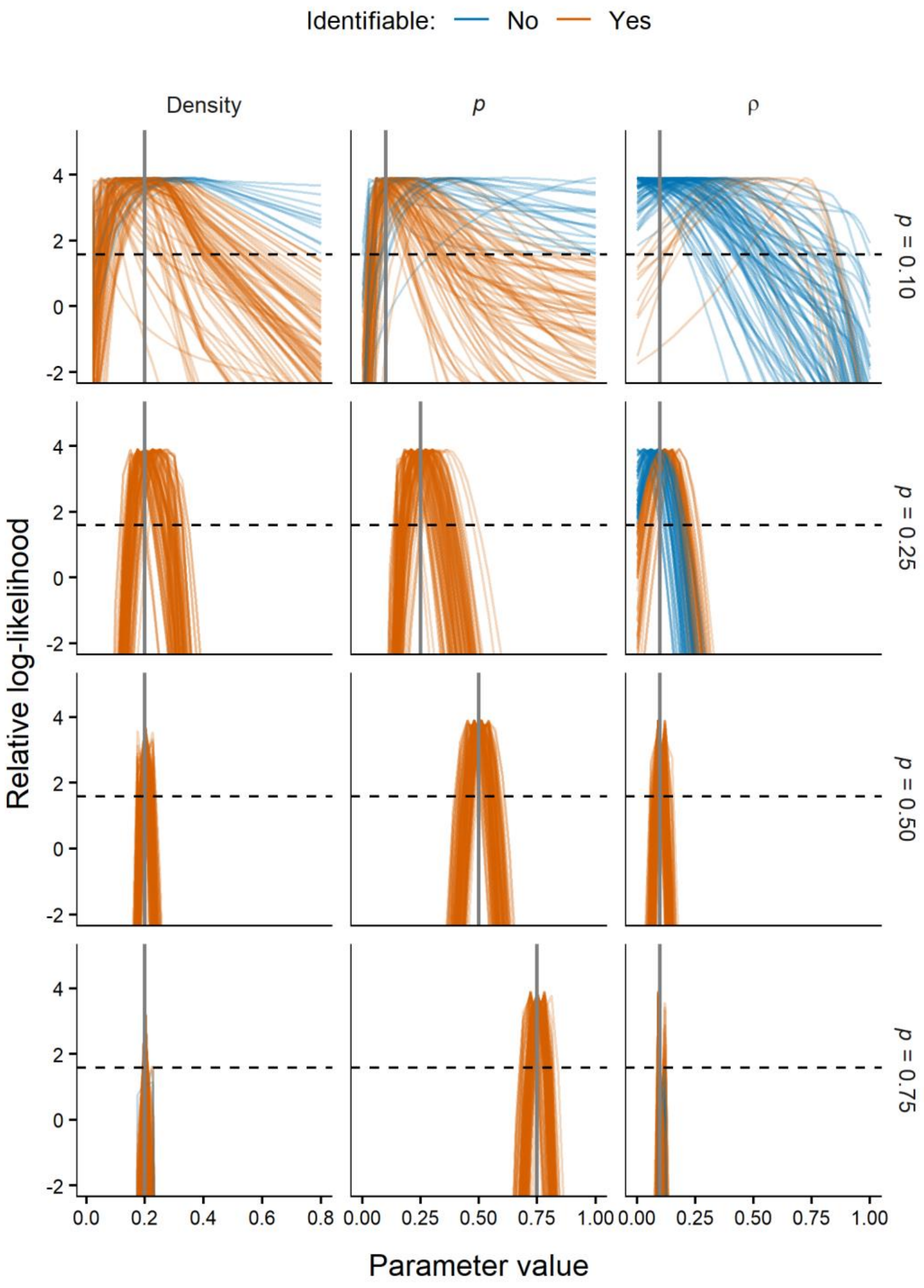

Figure S2. Likelihood profiles for 100 randomly selected data sets generated from a model with constant ρ for J = 6 removal samples. The dashed line represents confidence-limit thresholds for α = 0.20. Vertical gray lines indicate the true parameter value used to simulate removal data.

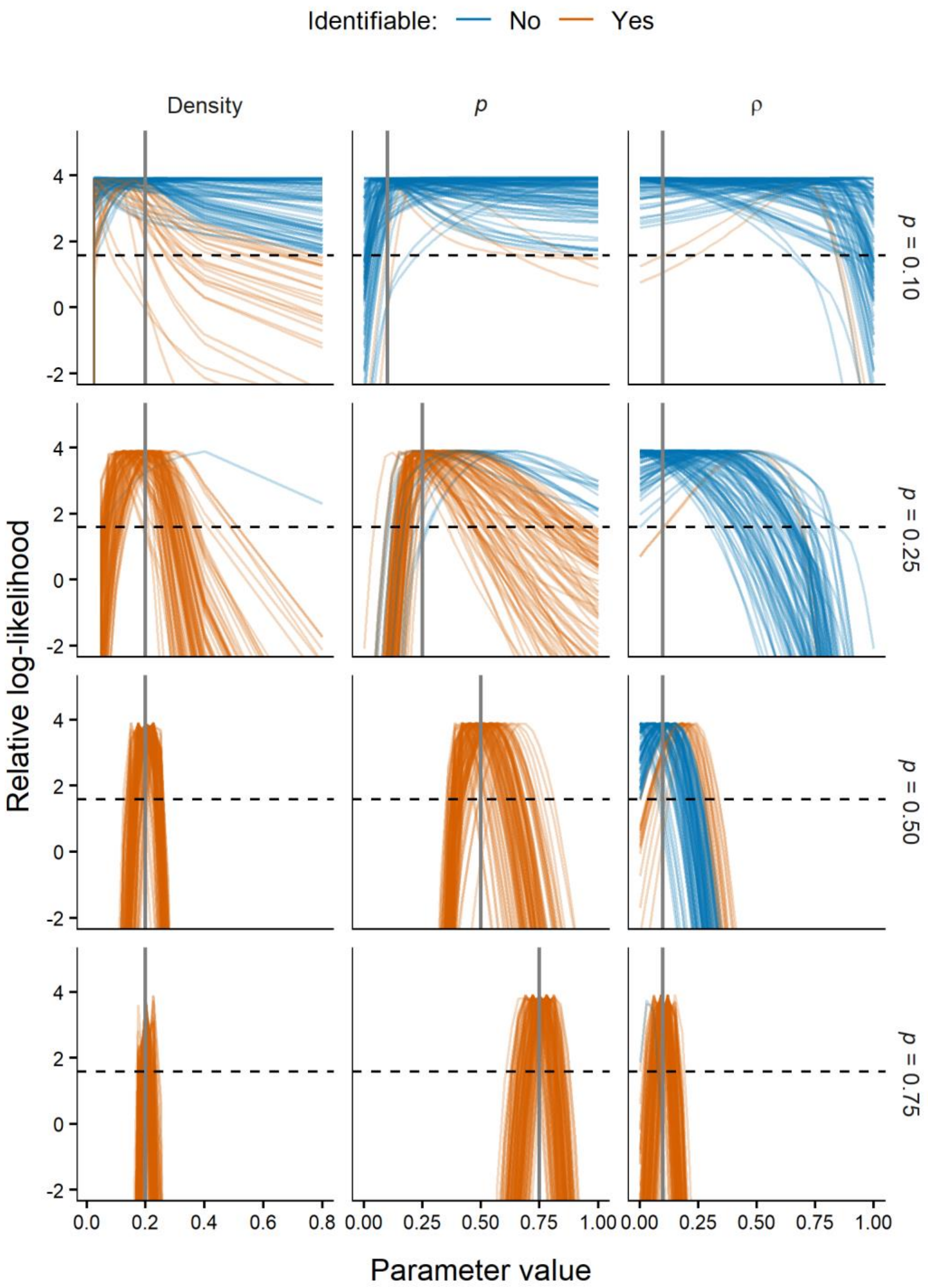

Figure S3. Likelihood profiles for 100 randomly selected datasets generated from a model with constant ρ for J = 3 removal samples. The dashed line represents confidence-limit thresholds for α = 0.20. Vertical gray lines indicate the true parameter value used to simulate removal data.

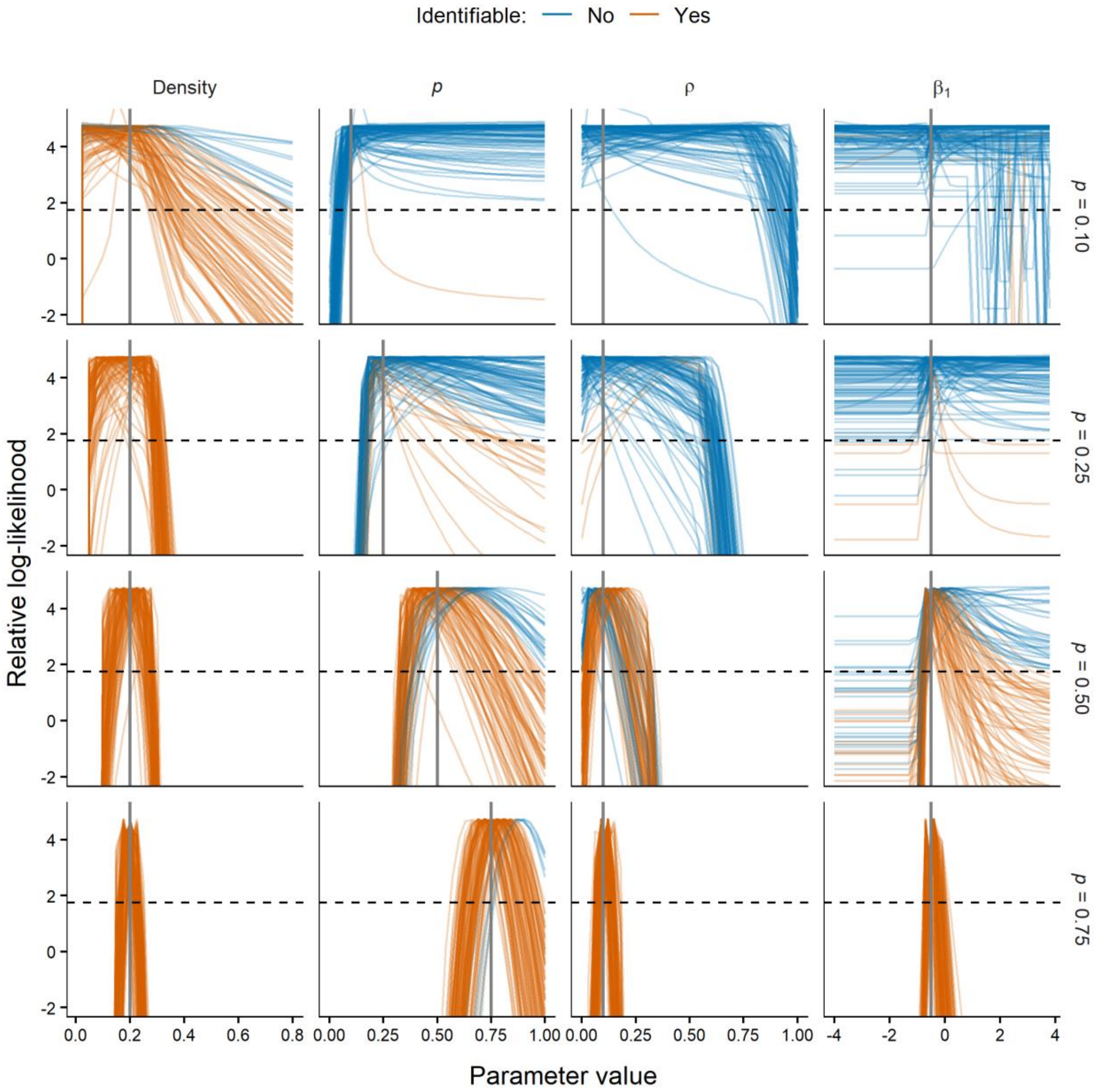

Figure S4. Likelihood profiles for 100 randomly selected datasets generated from a model with ρ declining as a function of sample occasion (*j*) for *J* = 6 removal samples. The dashed line represents confidence-limit thresholds for $\alpha = 0.20$. Vertical gray lines indicate the true parameter value used to simulate removal data.

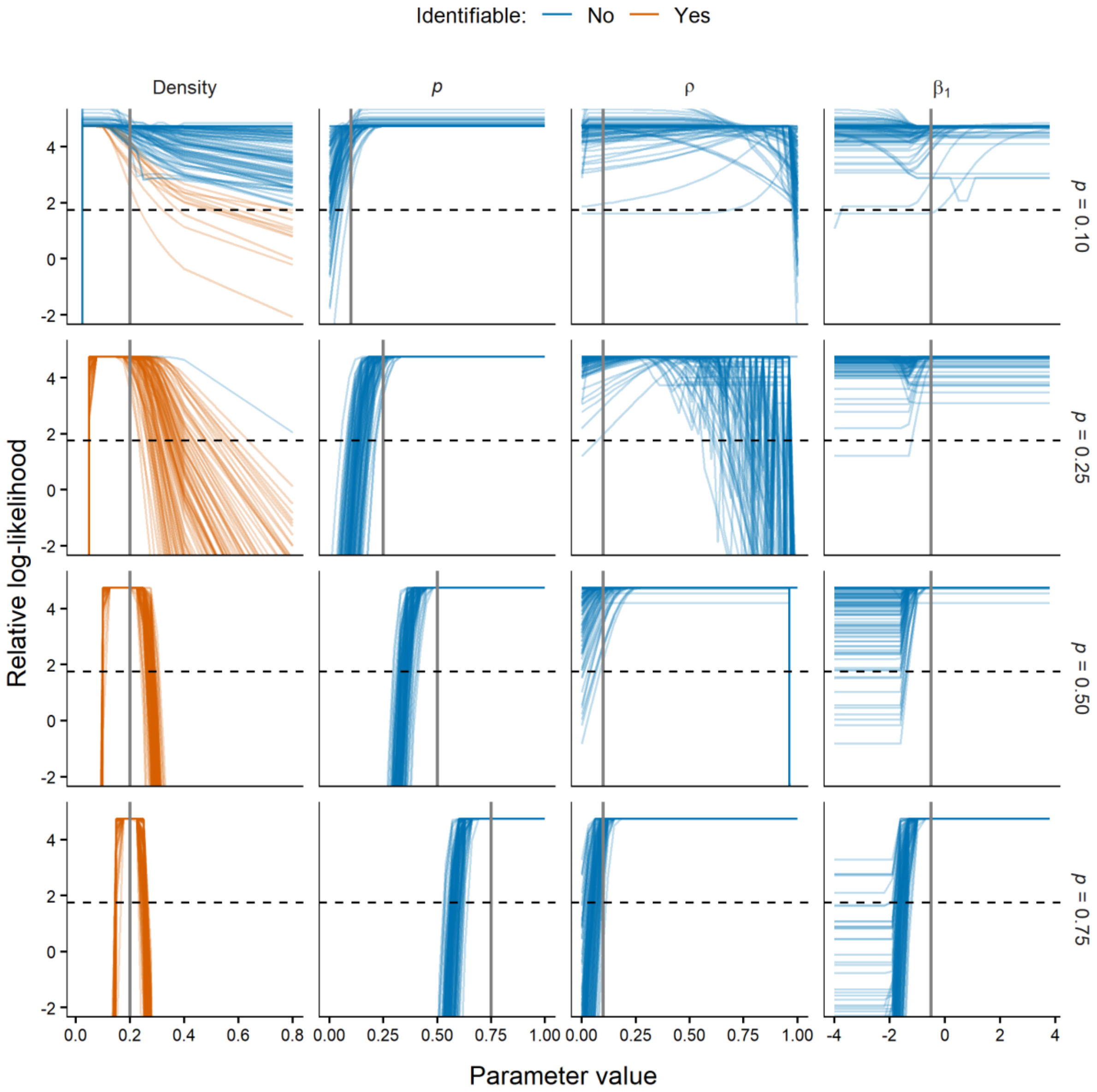

Figure S5. Likelihood profiles for 100 randomly selected datasets generated from a model with ρ declining as a function of sample occasion (j) for J = 3 removal samples. The dashed line represents confidence-limit thresholds for α = 0.20. Vertical gray lines indicate the true parameter value used to simulate removal data.

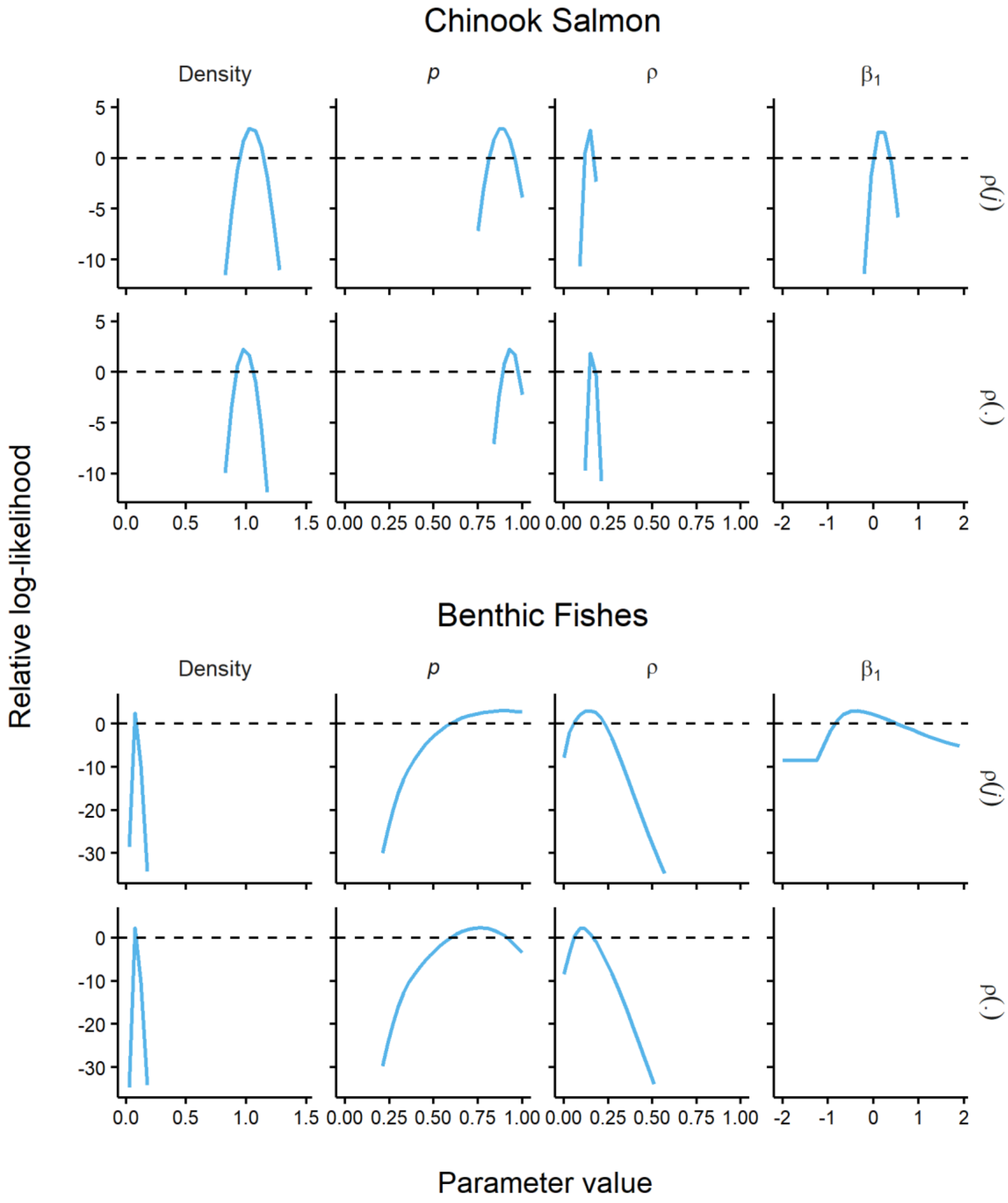

Figure S6. Likelihood profiles for case study data sets where removal sampling was performed using beach seines in the Sacramento-San Joaquin River Delta, USA. The dashed line represents confidence-limit thresholds for $\alpha = 0.20$. Each row represents a model with either a constant ρ (labeled as $\rho(.)$) or a model where ρ was expressed as a logit-linear function of sampling occasion (labeled as $\rho(j)$).
